# Correction to “SH2*scan*: Mapping SH2 Domain-Ligand Binding Selectivity for Inhibitors and Degraders”

**DOI:** 10.1021/acs.jmedchem.6c01212

**Published:** 2026-04-22

**Authors:** Luis M. Gonzalez Lira, Jennifer K. Wolfe-Demarco, Alexander M. Clifford, Tuan D. Le, Ghadeer M. Khasawneh, Medhanie Kidane, Michelle Nguyen, Julia A. Najera, Gabriel Pallares, Nicole B. Servant, Jean A. Bernatchez

The authors would like to add to the Acknowledgments section of the manuscript. The revised Acknowledgment is below.

