# Supplementary material for: SH2scan: Mapping SH2 Domain-Ligand Binding Selectivity for Inhibitors and Degraders
Source: J Med Chem. 2026 Jan 16;69(4):3932–40. doi: 10.1021/acs.jmedchem.5c02613 (PMC12951562; doi:10.1021/acs.jmedchem.5c02613)
Supplement: Supplementary file 1 [file jm5c02613_si_001.pdf]

# Supporting Information

# SH2*scan*: Mapping SH2 domain-ligand binding selectivity for inhibitors and degraders

Luis M. Gonzalez Lira[1,†], Jennifer K. Wolfe-Demarco[1,†], Alexander M. Clifford[1], Tuan D. Le[1], Ghadeer M. Khasawneh[1], Medhanie Kidane[1], Michelle Nguyen[1], Julia A. Najera[1], Gabriel Pallares[1], Nicole B. Servant[1], Jean A. Bernatchez[1*]

[1]Eurofins DiscoverX, LLC, 11180 Roselle Street, Suite D, San Diego, CA 92121, United States of America
†Luis M. Gonzalez Lira and Jennifer K. Wolfe-Demarco contributed equally to this work.
[*]To whom correspondence should be addressed: Jean A. Bernatchez, e-mail address: Jean.Bernatchez@discovery.eurofinsus.com

**Contents of Supporting Information**

Supporting Figure 1. Chemical characterization information for the test competitor compounds in this study. (S2)
Supporting Table 1. SH2*scan* protein construct details. (S39)
Supporting Table 2. Primary screening data for the 9 compounds tested in SH2*scan*. (S42)
Supporting Table 3. Replicate numbers for primary screening data for the 9 compounds tested in SH2*scan*. (S46)
Supporting Table 4. Dissociation constant values for the 9 compounds tested in SH2*scan*. (S49)
Supporting Table 5. Replicate numbers for dissociation constant values for the 9 compounds tested in SH2*scan*. (S52)
Supporting Table 6. Literature SH2 domain binding measurements for compounds relevant to this study. (S55)
Supporting Table 7. The allosteric inhibitors JAB-3312 and TNO155 do not compete with the phosphopeptide capture ligand for either of the PTPN11 SH2 domain-containing constructs in SH2*scan*. (S56)
Supporting Table 8. $K_D$ values for the nonbiotinylated forms of capture ligands and optimized bead loading percentages for each assay in SH2*scan*. (S57)

**Supporting Figure 1. Chemical characterization information for the test competitor compounds in this study.** Shown below is the information pertaining to chemical analysis for CGP78850, MN551, SD-36, SI-109, Stafib-1, STAT6-IN-1, STAT6-IN-3, JAB-3312 and TNO155.

$^1H$ NMR for CGP78850.

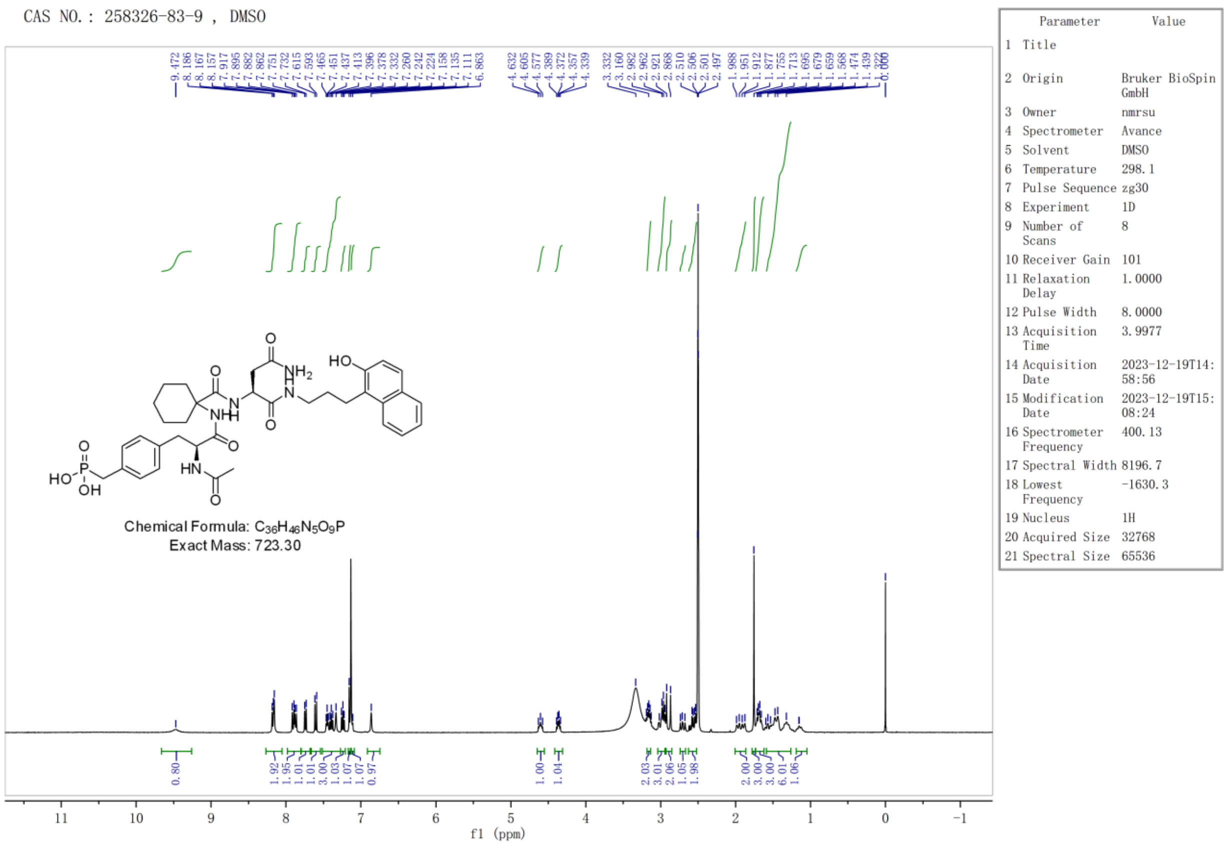

LCMS for CGP78850.

LC/MS Report

File E:\ChemStation\1\DATA\2023\2023-12\2023-12-19\BIZ2023-D19-SCH22-UPLC-MS-10-022502.D
Injection Date : 19-Dec-23, 18:28:54 Tgt Mass(EZX) :
Sample Name : BIZ2023-D19-SCH22 Location : P2-D-01
Acq. Operator : LY_2278 Inj : 1
Spec. Reported : MS Integration Inj Volume : 0.8 ul
Acq. Method : E:\ChemStation\1\Methods\1-POS-UPLC3.0MIN(1.2)(150-1000).M
Analysis Method : E:\CHEMSTATION\1\METHODS\1-POS-UPLC3.0MIN(1.2)(150-1000).M
CAS NO. : 258326-83-9 WalkUp method: '1-POS-3MIN' Project: BIZ2023
Method Info : Mobile Phase: A: water(0.01%TFA) B:ACN(0.01%TFA)
Gradient: 5% to 95%B within 2.0 min
Flow Rate :1.2ml/min
Column :Shim-pack Scepter C18-120, 3.0*33mm,3um
Oven Temperature : 45C

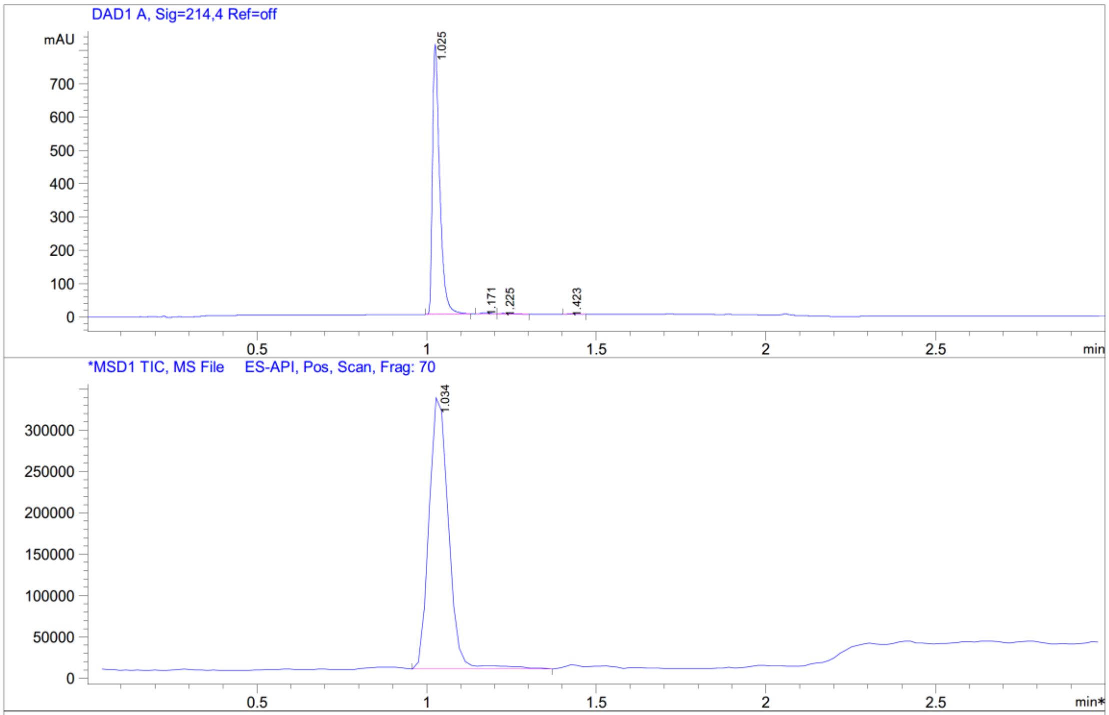


Integration Results for DAD1 A, Sig=214,4 Ref=off

| RetTim | Width | Area | Height | Area% |
|---|---|---|---|---|
| 1.03 | 0.02 | 1196.97 | 810.83 | 99.33 |
| 1.17 | 0.02 | 5.00 | 3.10 | 0.41 |
| 1.23 | 0.03 | 1.63 | 0.91 | 0.14 |
| 1.42 | 0.02 | 1.50 | 0.98 | 0.12 |

Integration Results for MSD1 TIC, MS File

| RetTim | Width | Area | Height | Area% |
|---|---|---|---|---|
| 1.03 | 0.06 | 1319991.88 | 338030.75 | 100.00 |

LC/MS Report

Ret. Time: 1.03 <<<< POSITIVE SPECTRA >>>>

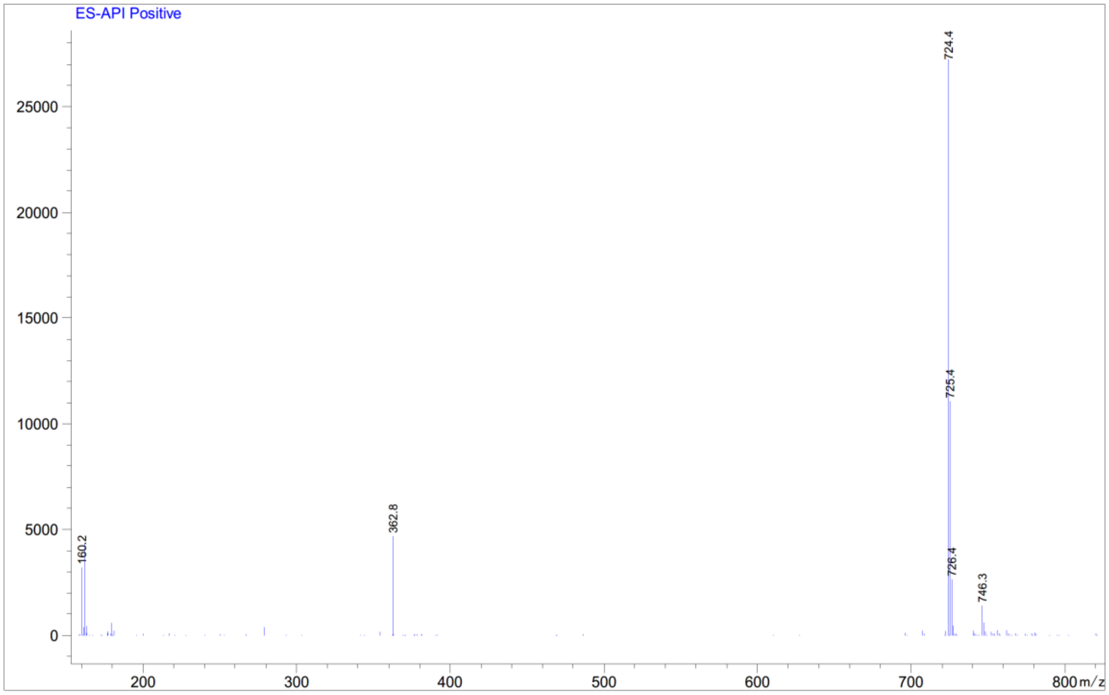

$^{1}$H NMR for MN551.

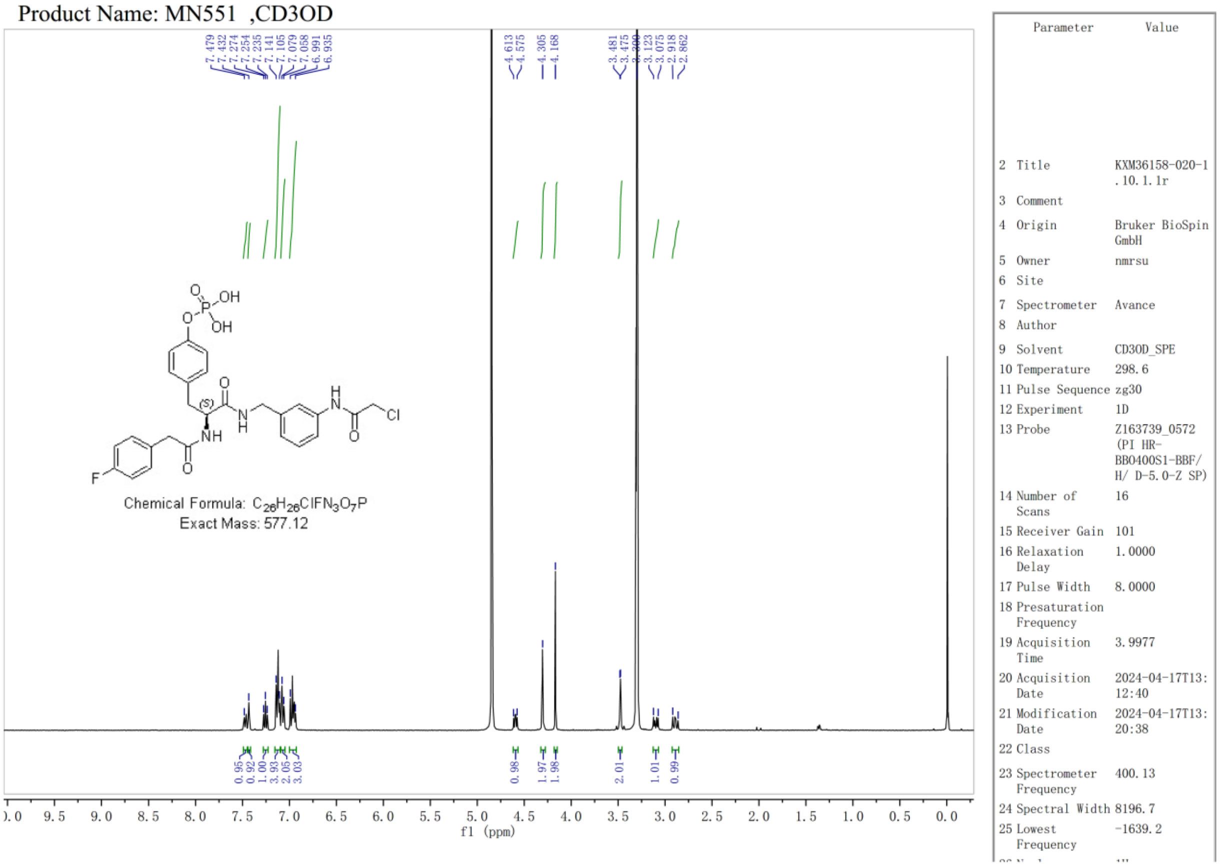

LCMS for MN551.

LC/MS Report

```
File D:\data\2024-04\0419-01 09-20-17\KXM36158-020-1B.D
Injection Date    : 19-Apr-24, 11:30:20             Tgt Mass(EZX)  :
Sample Name       : KXM36158-020-1B                            Location :
Acq. Operator     : SYSTEM                               Inj : 1
Spec. Reported    : MS Integration                    Inj Volume : 2 ul
Acq. Method       : D:\data\2024-04\0419-01 09-20-17\C2_4min_5_95A_50_800.M
Analysis Method   : D:\data\2024-04\0419-01 09-20-17\C2_4min_5_95A_50_800.M
Product Name: MN551    Sample Info : 577.93
Method Info : Instrument: LCMS005
              Mobile Phase: A: 0.1% acid B: ACN
              Gradient: 5% to 95% within 1.3min
              Flow Rate: 1.2mL/min
              Column: 4.6*50mm,3.5um YTA-RP-18
              Oven Temperature: 40C
```

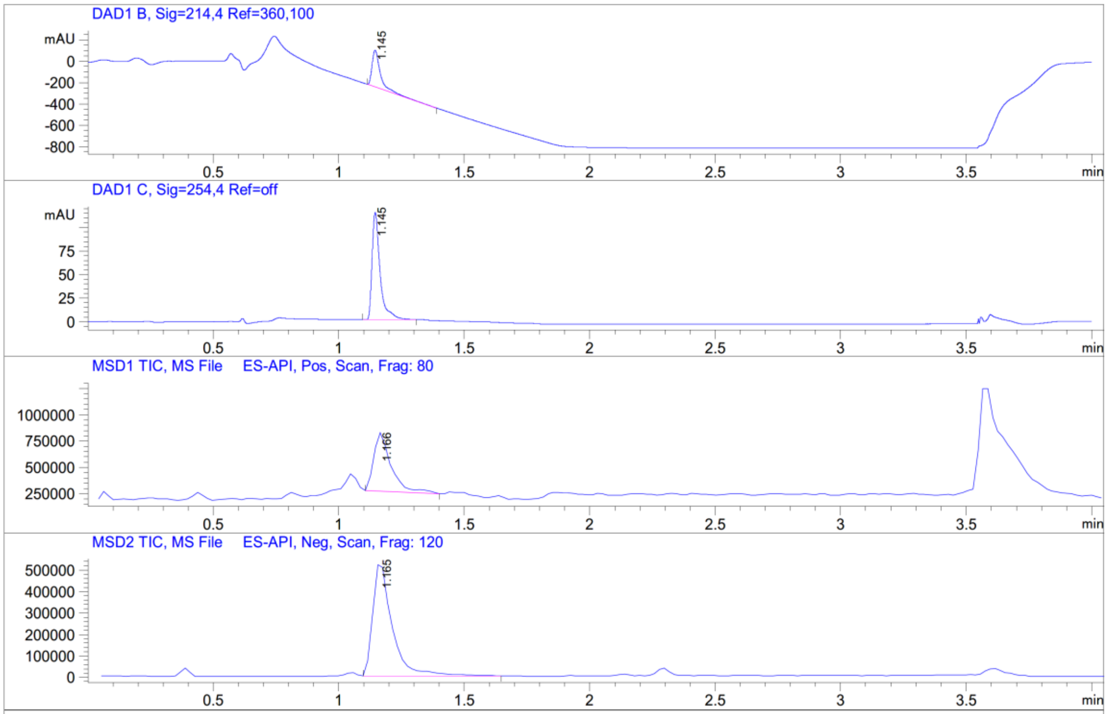


Integration Results for DAD1 B, Sig=214,4 Ref=360,100

| RetTim | Width | Area | Height | Area% |
|---|---|---|---|---|
| 1.14 | 0.03 | 778.32 | 344.42 | 100.00 |

Integration Results for DAD1 C, Sig=254,4 Ref=off

| RetTim | Width | Area | Height | Area% |
|---|---|---|---|---|
| 1.15 | 0.03 | 252.58 | 113.81 | 100.00 |

Integration Results for MSD1 TIC, MS File

| RetTim | Width | Area | Height | Area% |
|---|---|---|---|---|
| 1.17 | 0.07 | 2707823.00 | 558271.88 | 100.00 |

Integration Results for MSD2 TIC, MS File

| RetTim | Width | Area | Height | Area% |
|---|---|---|---|---|

| | | | | |
|---|---|---|---|---|
| 1.16 | 0.08 | 2846208.75 | 543329.50 | 100.00 |

Ret. Time: 1.17 <<<< POSITIVE SPECTRA >>>>

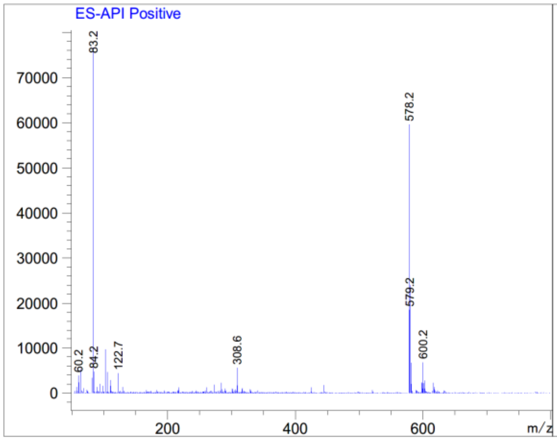


Ret. Time: 1.16 <<<< NEGATIVE SPECTRA >>>>

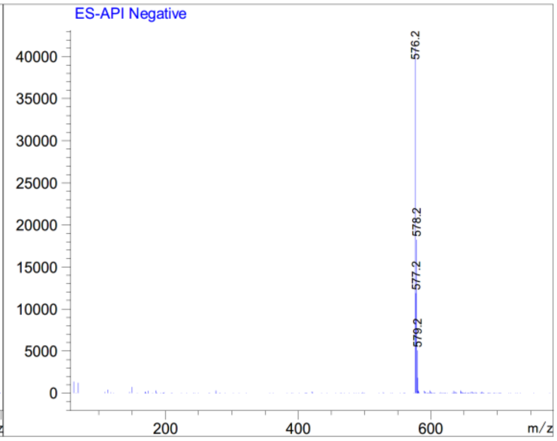

RP-HPLC for MN551.

# Analysis Report

<Sample Information>

Sample Name : KXM36158-020-1A
Method Filename : 20-80A(0-8)15min.lcm
Vial # : 1-33
Inj. Volume : 15 uL
Date Acquired : 2024-04-18 12:41:00

Product Name : MN551
Instrument : daojin001
Acquired by : System Administrator

<Chromatogram>

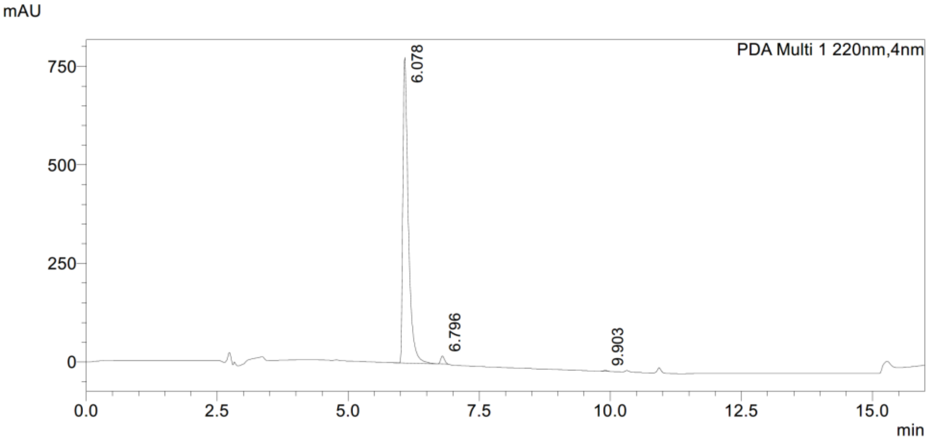


<Peak Table>

PDA Ch1 220nm

| Peak# | Ret. Time | Area | Height | Height% | Area% |
|---|---|---|---|---|---|
| 1 | 6.078 | 5623351 | 775315 | 97.178 | 98.055 |
| 2 | 6.796 | 101991 | 20276 | 2.541 | 1.778 |
| 3 | 9.903 | 9576 | 2238 | 0.280 | 0.167 |
| 总计 | | 5734918 | 797829 | 100.000 | 100.000 |

$^1H$ NMR for SD-36.

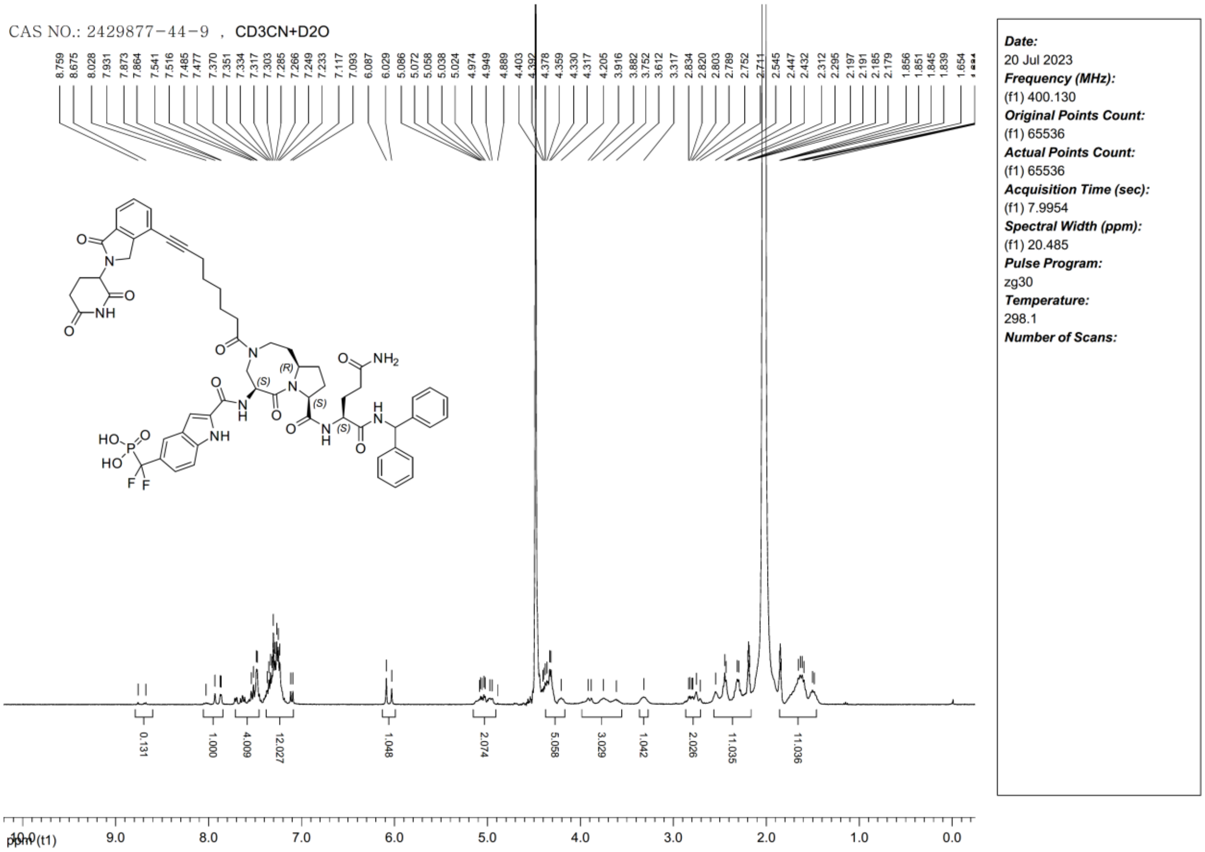

LCMS for SD-36.

LC/MS Report

**File F:\data\2023\2023-07\2023-07-19\WLF26880-001-8-1-UPLC-MS-05-013878.D**
**Injection Date : 19-Jul-23, 13:49:35 Tgt Mass(EZX) :**
**Sample Name : WLF26880-001-8-1 Location : P2-D-01**
**Acq. Operator : LY_2278 Inj : 1**
**Spec. Reported : MS Integration Inj Volume : 1 ul**
**Acq. Method : D:\methods\1-POS-UPLC1.4MIN(1.2)(0-2000).M**
**Analysis Method : D:\METHODS\1-POS-UPLC1.4MIN(1.2)(0-2000).M**
**CAS NO. : 2429877-44-9 WalkUp method: '1-POS-1.4MIN(0-2000)' Project: LD1872**
**Method Info : Mobile Phase: A: water(0.01%TFA) B:ACN(0.01%TFA)**
**Gradient: 5% to 95%B within1.2 min**
**Flow Rate :1.2ml/min**
**Column :Shim-pack Scepter C18-120, 3.0*33mm,3um**
**Oven Temperature : 45C**

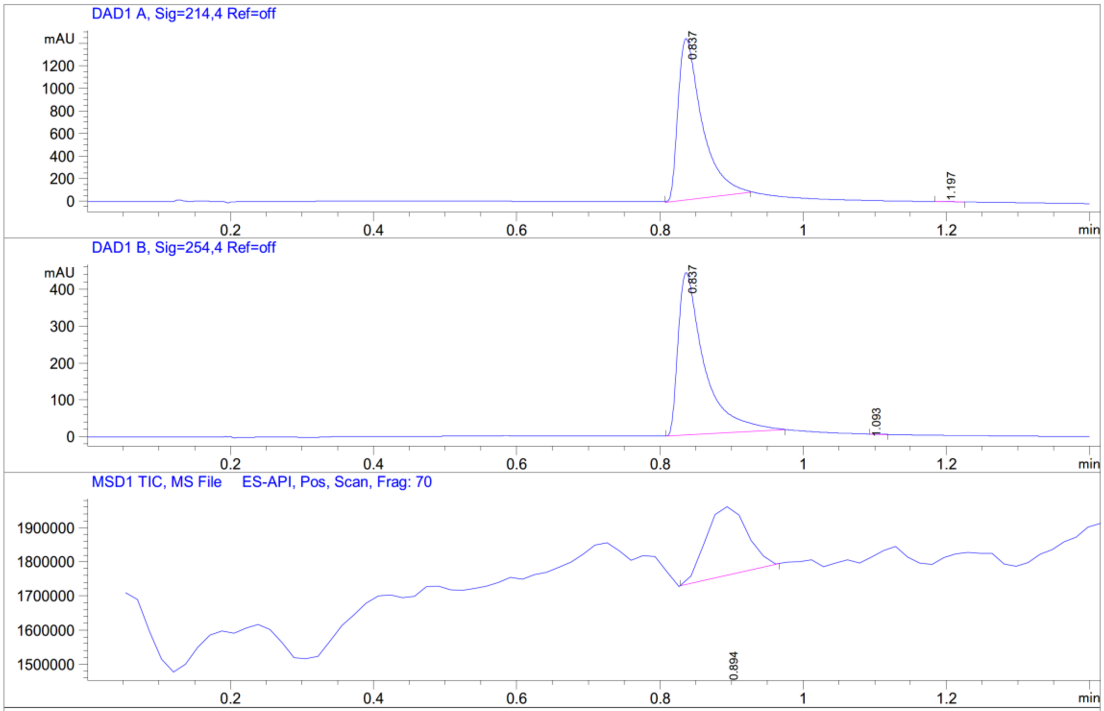


Integration Results for DAD1 A, Sig=214,4 Ref=off

| RetTim | Width | Area | Height | Area% |
|---|---|---|---|---|
| 0.84 | 0.04 | 3344.52 | 1432.56 | 99.91 |
| 1.20 | 0.02 | 3.12 | 2.67 | 0.09 |

Integration Results for DAD1 B, Sig=254,4 Ref=off

| RetTim | Width | Area | Height | Area% |
|---|---|---|---|---|
| 0.84 | 0.04 | 1115.79 | 440.08 | 99.96 |
| 1.09 | 0.02 | 0.50 | 0.20 | 0.04 |

Integration Results for MSD1 TIC, MS File

| RetTim | Width | Area | Height | Area% |
|---|---|---|---|---|
| 0.89 | 0.06 | 797361.00 | 200137.73 | 100.00 |

Ret. Time: 0.89 <<<< POSITIVE SPECTRA >>>>

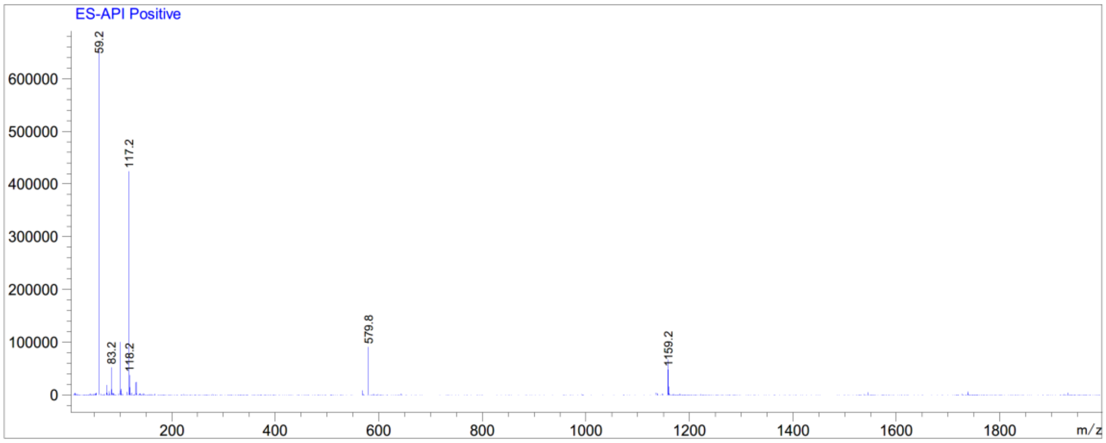

RP-HPLC for SD-36.



## Chromatogram and Results

### Injection Details

| | | | |
|---|---|---|---|
| *Injection Name:* | **WLF26880-001-8-1** | *Run Time (min):* | **25.00** |
| *Vial Number:* | **GE3** | *Injection Volume:* | **12.00** |
| *Injection Type:* | **Unknown** | *Channel:* | **UV_VIS_1** |
| *CAS NO.:* | **2429877-44-9 A-RP-1464** | *Wavelength:* | **210** |
| *Instrument Method:* | **HM-713F_17-H01P01(RP-HPLC).M** | *Bandwidth:* | **4** |
| *Processing Method:* | **3D Quantitative** | *Dilution Factor:* | **1.0000** |
| *Injection Date/Time:* | **20/Jul/23 10:51** | seq.update_operator | **jzq** |
| seq.creation_operator | **jzq** | instMeth.creation_operator | **jlw** |
| injection.dilution_factor | **1.0000** | rdf.update_operator | **jzq** |
| instMeth.creation_time | **09/Jan/2023 13:04** | | |
| chm.rawpath | **chrom://hy557/DATA/Instrument Data/202307/20230720.seq/489.smp/UV_VIS_1.channel/UV_VIS_1.chm** | | |

### Chromatogram

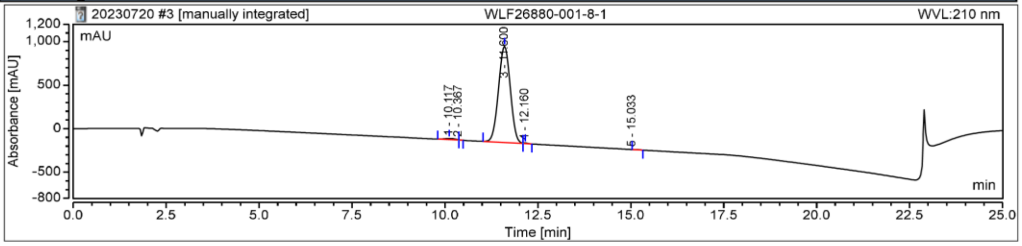


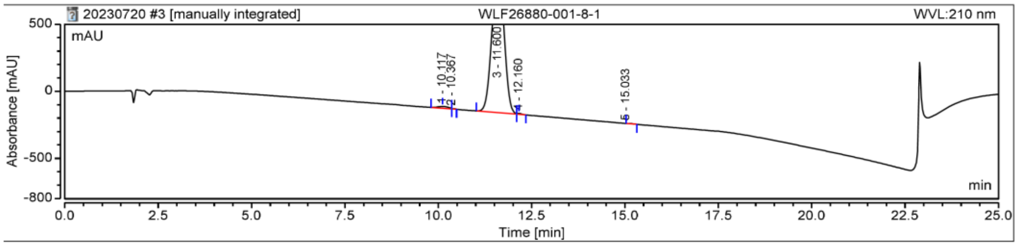


### Integration Results

| No. | Peak Name | Retention Time min | Area mAU*s | Height mAU | Relative Area % | Resolution (USP) | Match | S/N |
|---|---|---|---|---|---|---|---|---|
| 1 | | 10.117 | 276.7669 | 13.880 | 1.1134 | n.a. | 821 | 2.3 |
| 2 | | 10.367 | 16.0147 | 4.015 | 0.0644 | n.a. | 554 | n.a. |
| 3 | | 11.600 | 24490.9596 | 1106.423 | 98.5221 | n.a. | 992 | 1389.2 |
| 4 | | 12.160 | 54.8991 | 7.488 | 0.2208 | 1.4 | 794 | n.a. |
| 5 | | 15.033 | 19.7005 | 0.000 | 0.0793 | n.a. | 1000 | n.a. |
| **Total:** | | | **24858.3408** | **1131.806** | **100.0000** | | **4160** | |

$^{1}$H NMR for SI-109.

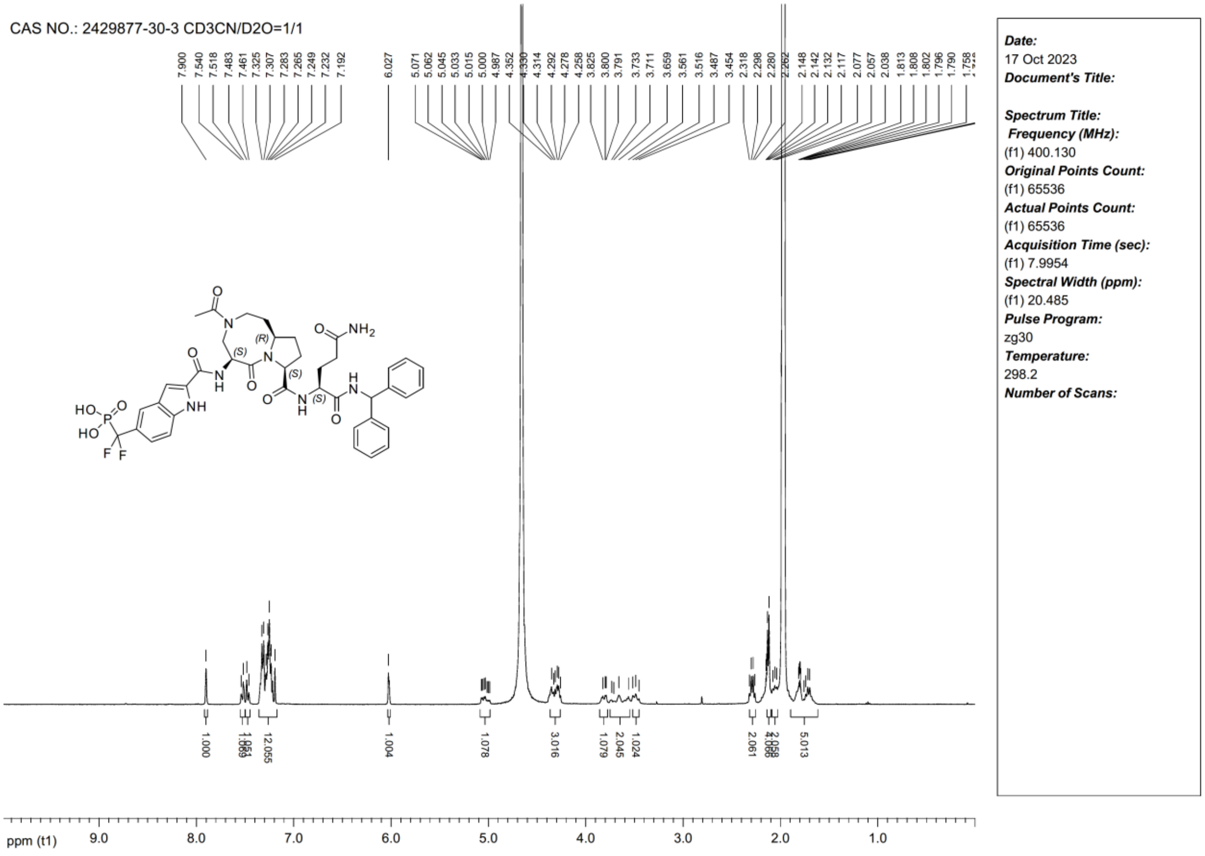

LCMS for SI-109.

LC/MS Report

File F:\data\2023\2023-10\2023-10-16\WLF31346-001-5A-UPLC-MS-05-023258.D
Injection Date : 16-Oct-23, 10:57:10 Tgt Mass(EZX) :
Sample Name : WLF31346-001-5A Location : P1-D-06
Acq. Operator : LY_2278 Inj : 1
Spec. Reported : MS Integration Inj Volume : 0.5 ul
Acq. Method : D:\methods\1-POS-UPLC2.0MIN(0.8)-(0-2000).M
Analysis Method : D:\METHODS\1-POS-UPLC2.0MIN(0.8)-(0-2000).M
CAS NO. : 2429877-30-3 WalkUp method: '1-POS-2MIN(0-2000)' Project: LD2037
Method Info : Mobile Phase: A: water(0.01%TFA) B:ACN(0.01%TFA)
Gradient: 5% to 95%B within 1.3 min
Flow Rate :0.8ml/min
Column :Poroshell 120 EC- C18, 2.1*50mm,1.9um
Oven Temperature : 45C

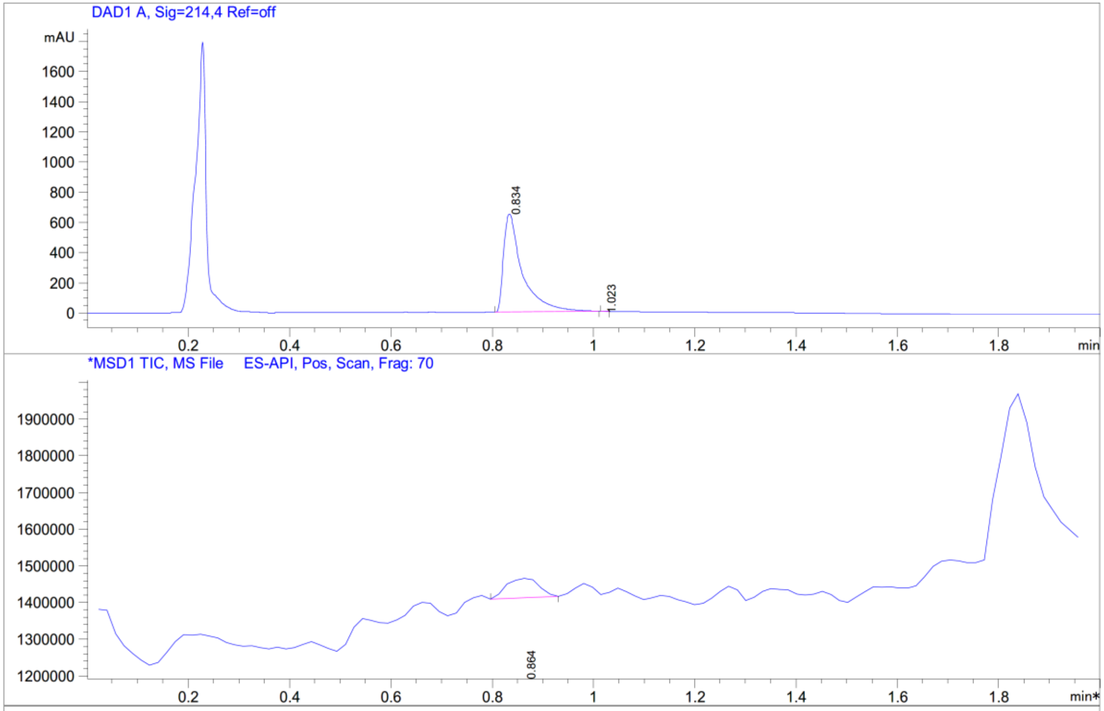


Integration Results for DAD1 A, Sig=214,4 Ref=off

| RetTim | Width | Area | Height | Area% |
|---|---|---|---|---|
| 0.83 | 0.04 | 1673.65 | 650.10 | 99.97 |
| 1.02 | 0.01 | 0.57 | 0.72 | 0.03 |

Integration Results for MSD1 TIC, MS File

| RetTim | Width | Area | Height | Area% |
|---|---|---|---|---|
| 0.86 | 0.07 | 236680.61 | 53098.68 | 100.00 |

LC/MS Report

Ret. Time: 0.86 <<<< POSITIVE SPECTRA >>>>

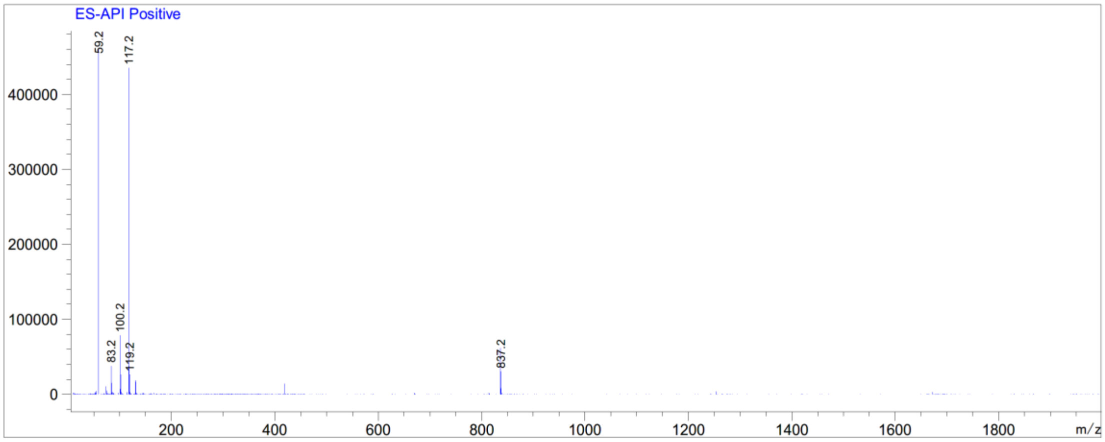

RP-HPLC for SI-109.

## SAMPLE INFORMATION

| | | | |
|---|---|---|---|
| CAS NO.: | 2429877-30-3 | Acquired By: | JZQ4027 |
| Sample Type: | Unknown | Sample Set Name: | 20231122 |
| Vial: | 2:F,5 | Instrument Method Name: | HM_713L_2_H01P02_RP_HPLC |
| Injection #: | 1 | Processing Method: | HPLC 118 |
| Injection Volume: | 5.00 ul | Channel Name: | 2998 Ch1 210nm@1.2nm |
| Run Time: | 25.0 Minutes | Label | A-RP-1283 |
| Date Acquired: | 11/22/2023 10:15:02 AM CST | Column Serial Number | |
| Date Processed: | 11/22/2023 11:15:58 AM CST | Processed By: | JZQ4027/group_leader |

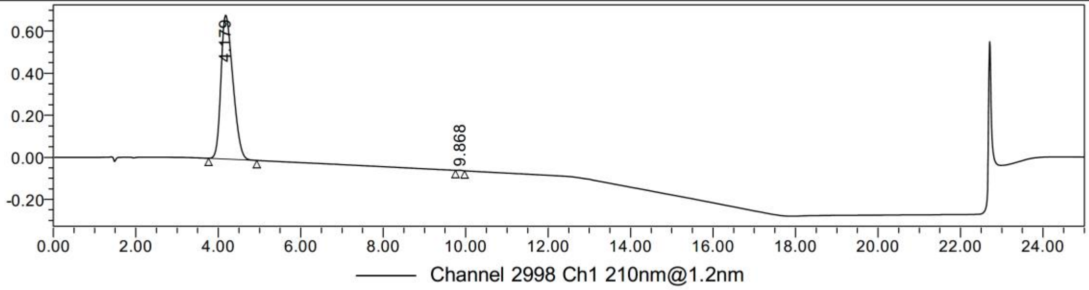


| | RT | Area | Width (sec) | Height | Resolution | % Area | Name |
|---|---|---|---|---|---|---|---|
| 1 | 4.179 | 13825462.2252 | 69.900 | 684087.415 | | 99.913 | |
| 2 | 9.868 | 12103.2205 | 13.200 | 1903.271 | 16.04 | 0.087 | |

Reported by User: JZQ4027 (JZQ4027)
Report Method: Report2
Report Method ID 3580
Page: 1 of 1

Project Name: HPLC-118\2023\202311
Date Printed:
11/22/2023
11:16:08 AM PRC

<sup></sup>

$^{1}$H NMR for Stafib-1.

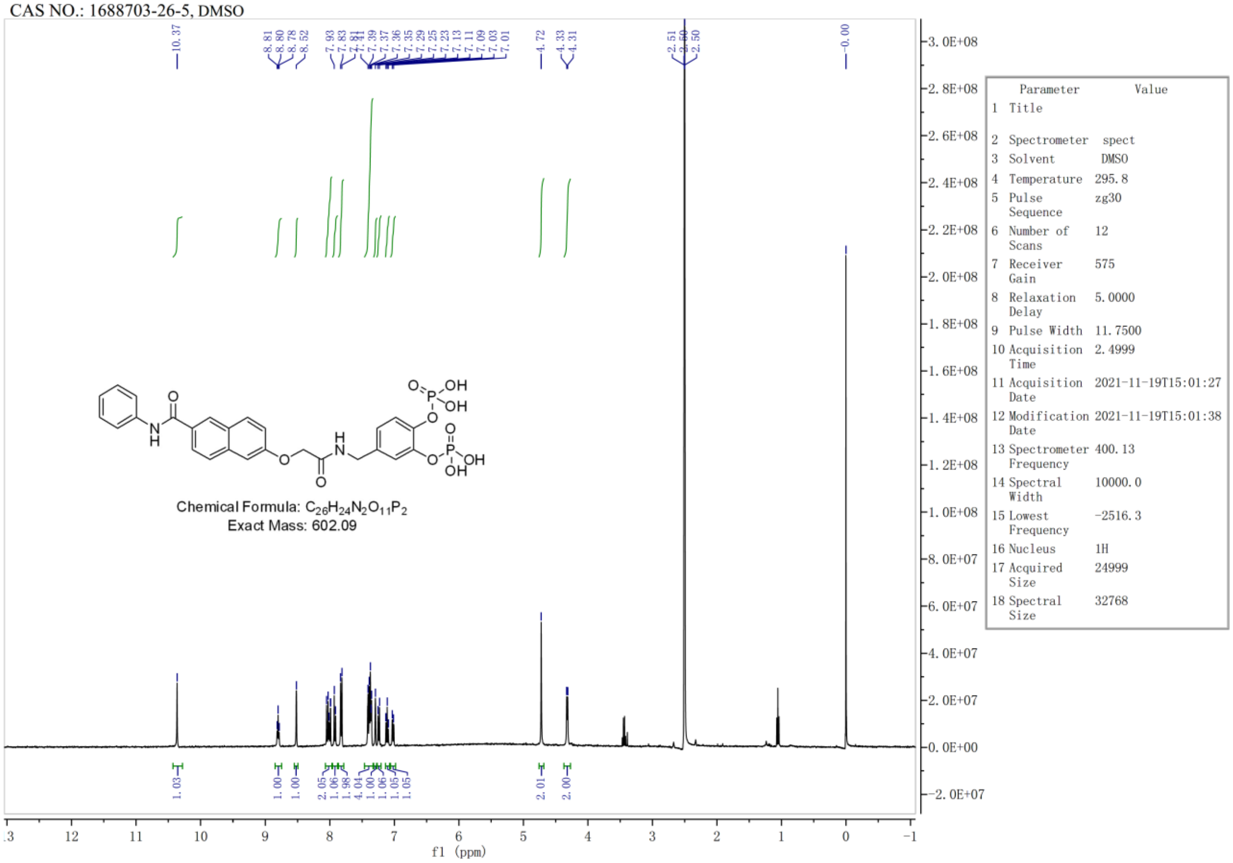

MS for Stafib-1.

CAS NO.: 1688703-26-5

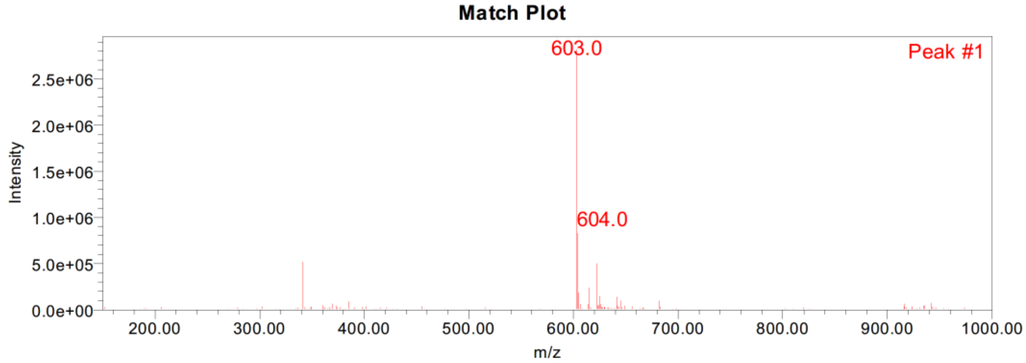


Retention Time 0.724 Channel Name MS TIC

RP-HPLC for Stafib-1.



## Chromatogram and Results

**Injection Details**

| | | | |
|---|---|---|---|
| *Injection Name:* | **BIZ2021-N11-WLQ5** | *Run Time (min):* | **16.00** |
| *Vial Number:* | **BA6** | *Injection Volume:* | **2.00** |
| *Injection Type:* | **Unknown** | *Channel:* | **UV_VIS_1** |
| *User:* | **HuXinQuan** | *Wavelength:* | **214** |
| *CAS NO.:* | **1688703-26-5 A-RP-541** | *Bandwidth:* | **4** |
| *Instrument Method:* | **10-90A,214NM** | *Dilution Factor:* | **1.0000** |
| *Processing Method:* | **3D Quantitative** | *Sample Weight:* | **1.0000** |
| *Injection Date/Time:* | **12/Nov/21 15:04** | | |

chm.ra **chrom://hy583/HPLC-63/202111/20211112.seq/417.smp/UV_VIS_1.channel/UV_VIS_1.chm**

**Chromatogram**

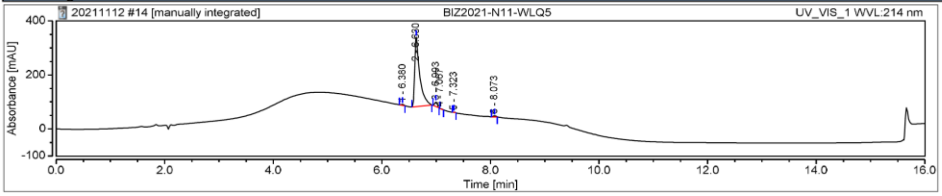


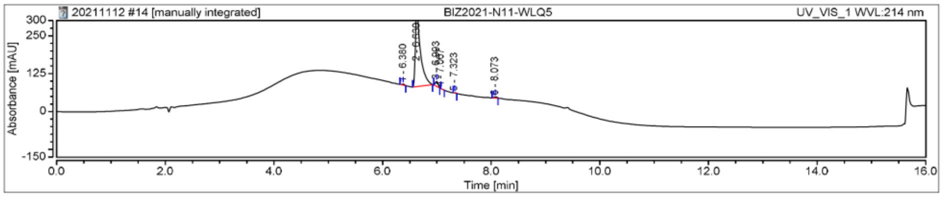


**Integration Results**

| No. | Peak Name | Retention Time min | Area mAU*min | Height mAU | Match | Resolution (SM) | Rel.Area % |
|---|---|---|---|---|---|---|---|
| 1 | | 6.380 | 0.158 | 3.460 | 926 | 1.58 | 0.55 |
| 2 | | 6.630 | 27.414 | 257.713 | 999 | 2.22 | 95.04 |
| 3 | | 6.993 | 0.951 | 16.888 | 999 | 1.13 | 3.30 |
| 4 | | 7.067 | 0.025 | 0.000 | 0 | 4.93 | 0.09 |
| 5 | | 7.323 | 0.053 | 1.076 | 868 | 9.99 | 0.18 |
| 6 | | 8.073 | 0.244 | 3.702 | 964 | n.a. | 0.85 |
| **Total:** | | | **28.845** | **282.838** | **4755.58** | **19.86** | |

$^{1}H$ NMR for STAT6-IN-1.

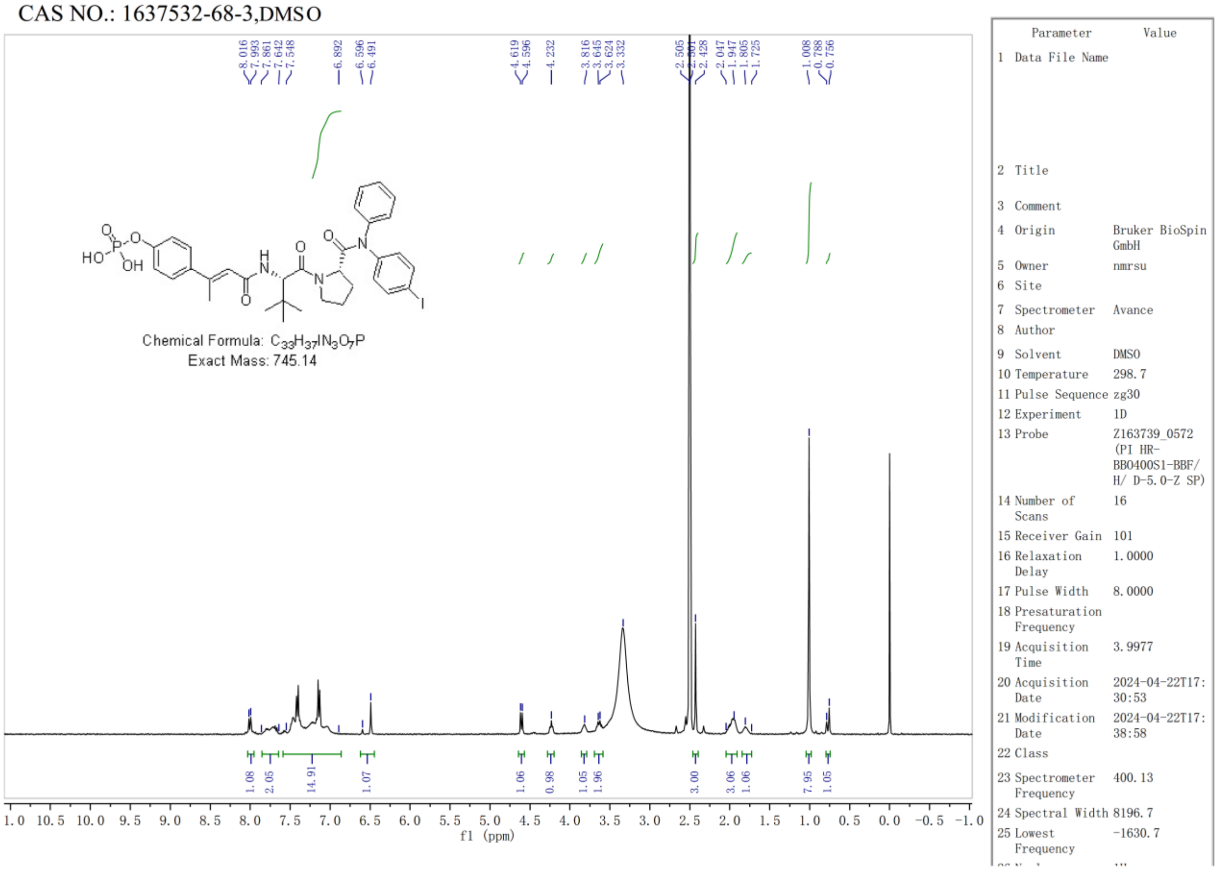

LCMS for STAT6-IN-1.

LC/MS Report

File D:\data\2024-04\0422-01 09-43-08\WJQ36125-021-2.D
Injection Date : 22-Apr-24, 15:24:17 Tgt Mass(EZX) :
Sample Name : WJQ36125-021-2 Location :
Acq. Operator : SYSTEM Inj : 1
Spec. Reported : MS Integration Inj Volume : 2 ul
Acq. Method : D:\data\2024-04\0422-01 09-43-08\C2_4min_5_95A_100_1200_UV.M
Analysis Method : D:\data\2024-04\0422-01 09-43-08\C2_4min_5_95A_100_1200_UV.M
CAS NO. : 1637532-68-3
Method Info : Instrument: LCMS005
Mobile Phase: A: 0.1% acid B: ACN
Gradient: 5% to 95% within 1.3min
Flow Rate: 1.2mL/min
Column: 4.6*50mm,3.5um YTA-RP-18
Oven Temperature: 40C

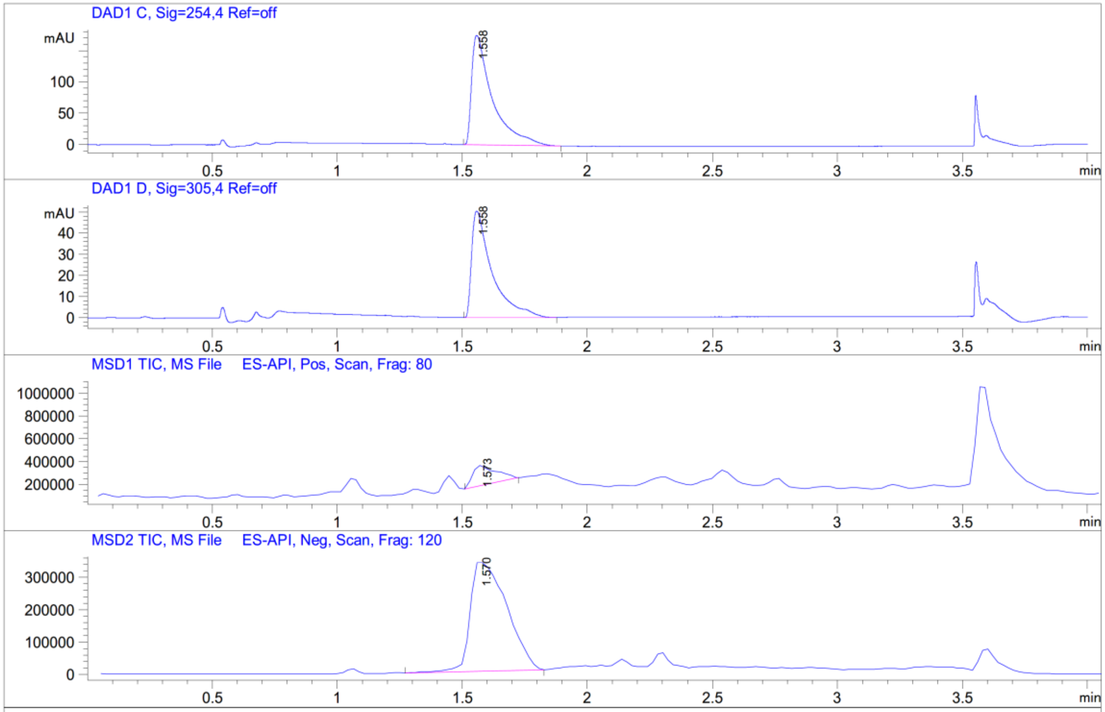


Integration Results for DAD1 C, Sig=254,4 Ref=off

| RetTim | Width | Area | Height | Area% |
|---|---|---|---|---|
| 1.56 | 0.08 | 1002.66 | 174.36 | 100.00 |

Integration Results for DAD1 D, Sig=305,4 Ref=off

| RetTim | Width | Area | Height | Area% |
|---|---|---|---|---|
| 1.56 | 0.08 | 285.63 | 50.41 | 100.00 |

Integration Results for MSD1 TIC, MS File

| RetTim | Width | Area | Height | Area% |
|---|---|---|---|---|
| 1.57 | 0.09 | 1143334.50 | 175487.92 | 100.00 |

Integration Results for MSD2 TIC, MS File

| RetTim | Width | Area | Height | Area% |
|---|---|---|---|---|

| | | | | |
|---|---|---|---|---|
| 1.57 | 0.15 | 3438094.75 | 348789.41 | 100.00 |

LC/MS Report

Ret. Time: 1.57  <<<< POSITIVE SPECTRA >>>>    Ret. Time: 1.57  <<<< NEGATIVE SPECTRA >>>>

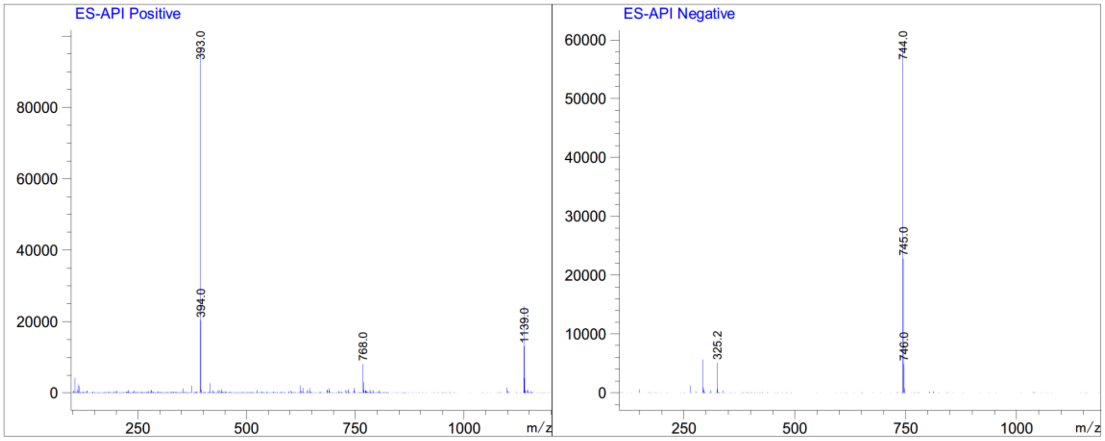

RP-HPLC for STAT6-IN-1.

# Analysis Report

| | | | |
|---|---|---|---|
| **CAS NO.:** | 1637532-68-3 | | |
| **Acq. method :** | 10-80(15min).M | | |
| **Location :** | P1-C4 | **Injection volume:** | 5.000 |
| **Injection date:** | 2024-04-22 15:57:45+08:00 | **Acq. operator :** | **1260** |

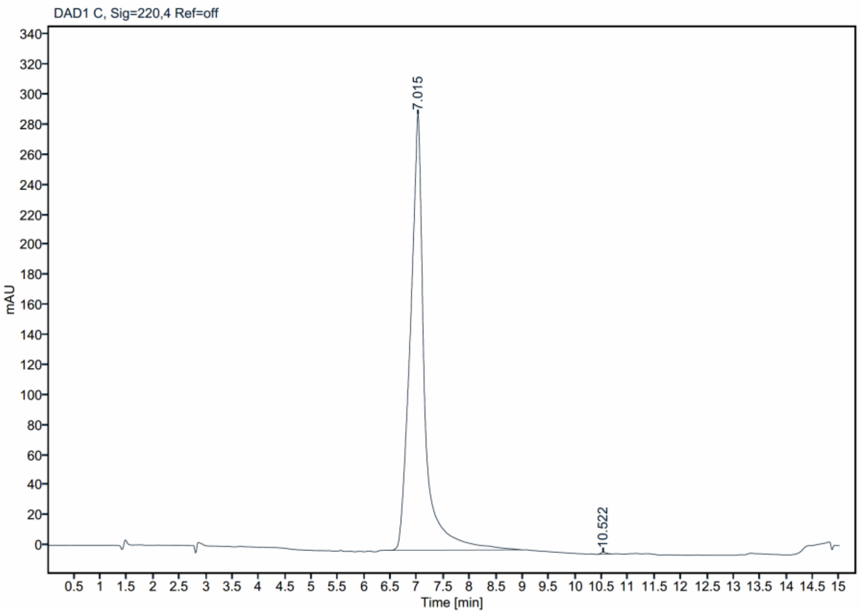


**Signal:** DAD1 C, Sig=220,4 Ref=off

| No. | RT [min] | Height | Height % | Area | Area% |
|---|---|---|---|---|---|
| 1 | 7.015 | 290.27 | 99.538 | 5399.56 | 99.818 |
| 2 | 10.522 | 1.35 | 0.462 | 9.86 | 0.182 |
| | Sum | 291.6147 | 100.00 | 5409.4169 | 100.0000 |

$^{1}$H NMR for STAT6-IN-3.

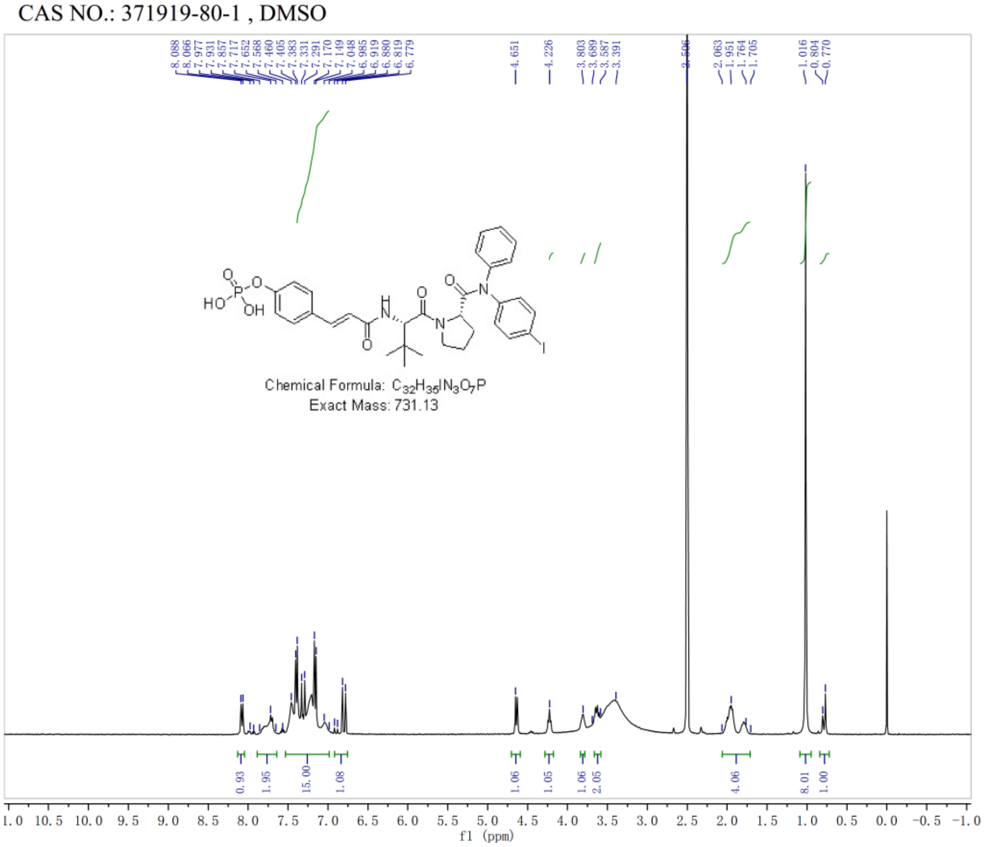


| Parameter | | Value |
|---|---|---|
| 1 | Data File Name | |
| 2 | Title | |
| 3 | Comment | |
| 4 | Origin | Bruker BioSpin GmbH |
| 5 | Owner | nmrsu |
| 6 | Site | |
| 7 | Spectrometer | Avance |
| 8 | Author | |
| 9 | Solvent | DMSO |
| 10 | Temperature | 298.9 |
| 11 | Pulse Sequence | zg30 |
| 12 | Experiment | 1D |
| 13 | Probe | Z163739_0572 (PI HR-BBO400S1-BBF/ H/ D-5.0-Z SP) |
| 14 | Number of Scans | 16 |
| 15 | Receiver Gain | 101 |
| 16 | Relaxation Delay | 1.0000 |
| 17 | Pulse Width | 8.0000 |
| 18 | Presaturation Frequency | |
| 19 | Acquisition Time | 3.9977 |
| 20 | Acquisition Date | 2024-04-24T12:43:47 |
| 21 | Modification Date | 2024-04-24T12:51:51 |
| 22 | Class | |
| 23 | Spectrometer Frequency | 400.13 |
| 24 | Spectral Width | 8196.7 |
| 25 | Lowest Frequency | -1630.6 |
| 26 | Nucleus | 1H |

LCMS for STAT6-IN-3.



# Analysis Report

## <Sample Information>

| | | | |
|---|---|---|---|
| CAS NO. | : 371919-80-1 | | |
| Sample Mw | : 731.51 | | |
| Data Filename | : WJQ39001-005-1.lcd | | |
| Method Filename | : C2-4min(5-95A)-(50-800)-1.2mLmin.lcm | | |
| Batch Filename | : 2024-0424-LCMS003-01.lcb | | |
| Vial # | : 1-8 | Instument | : LCMS-003 |
| Injection Volume | : 3 uL | | |
| Date Acquired | : 2024/04/24 10:09:28 | Acquired by | : System Administrator |
| Date Processed | : 2024/04/24 10:14:57 | Processed by | : System Administrator |

## <Chromatogram>

Chromatogram

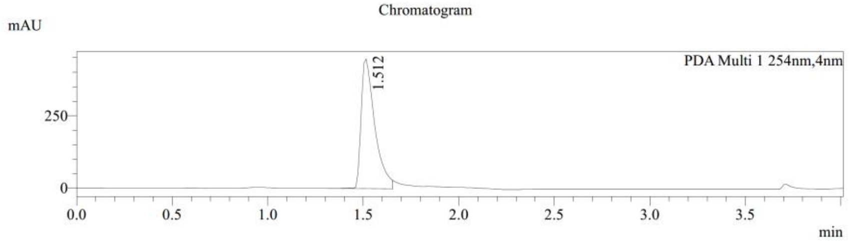


Peak Table

PDA Ch1 254nm

| Peak# | Ret. Time | Area | Height | Area% |
|---|---|---|---|---|
| 1 | 1.512 | 2238419 | 448175 | 100.000 |
| | | 2238419 | 448175 | 100.000 |

MS Chromatogram

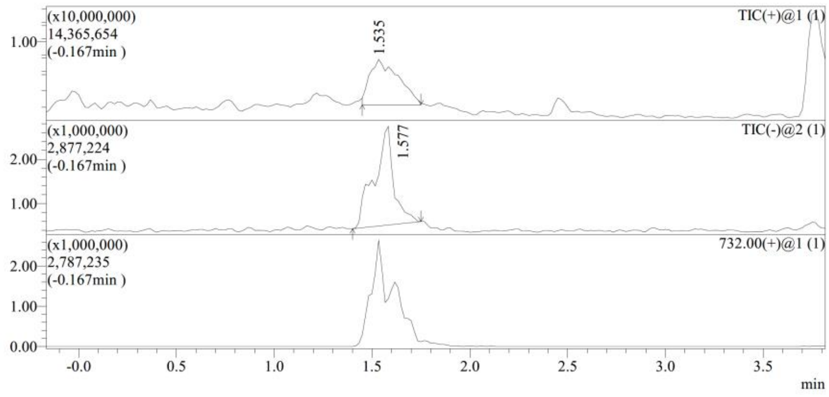


MASS Peak Table TIC

| Peak# | Ret. Time | Area | m/z | A/H | Event# | Area% |
|---|---|---|---|---|---|---|
| 1 | 1.535 | 56492185 | TIC | 10.153 | 1-1 | 100.000 |
| 2 | 1.577 | 15387733 | TIC | 6.906 | 1-2 | 100.000 |
| | | 71879918 | | | | 200.000 |

D:\LCMS\WJQ39001-005-1.lcd

MS Spectrum

Peak No:1 Event:1
Line#:1 R.Time:----(Scan#:----)
$If$(SpPrTab==SpPrTab)Spectrum Mode:Averaged 1.517-1.550(203-207)
Retention time:1.550
Base Peak:732.1(1891918)
TIC:(+)

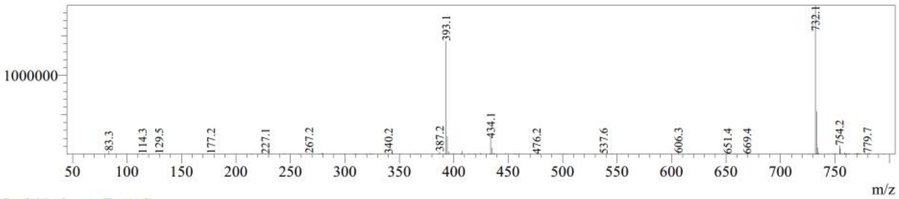


Peak No:2 Event:2
Line#:2 R.Time:----(Scan#:----)
$If$(SpPrTab==SpPrTab)Spectrum Mode:Averaged 1.558-1.591(208-212)
Retention time:1.591
Base Peak:730.2(1500949)
TIC:(-)

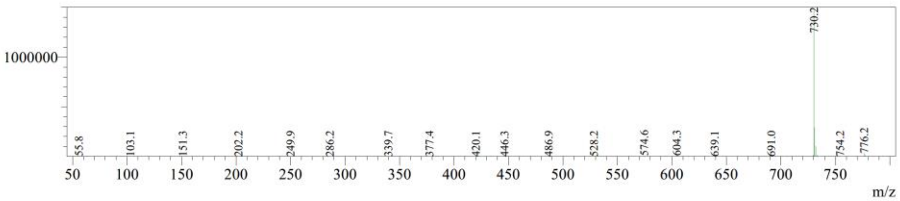

RP-HPLC for STAT6-IN-3.

# Analysis Report

## <Sample Information>

| | | | |
|---|---|---|---|
| CAS NO. | : 371919-80-1 | | |
| Method Filename | : 10-80B(15MIN).lcm | | |
| Vial # | : 1-19 | Instrument | : daojin004 |
| Injection Volume | : 10 uL | | |
| Date Acquired | : 2024-04-24 10:20:28 | Acquired by | : System Administrator |

## <Chromatogram>

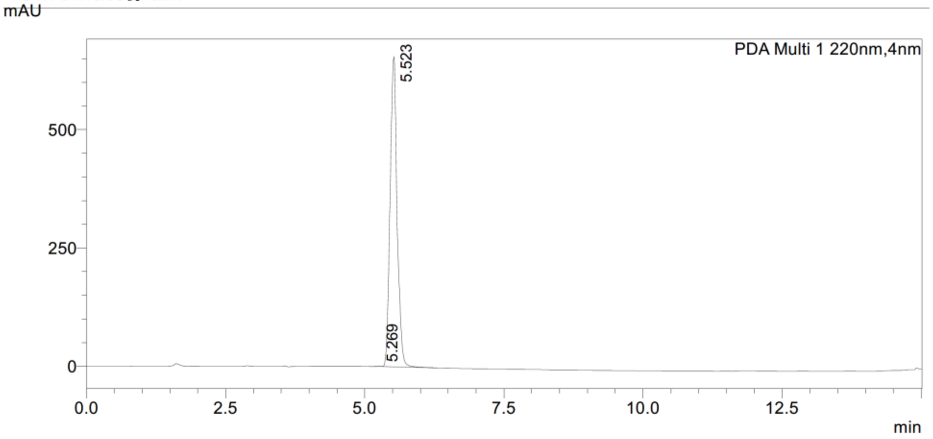


## <Peak Table>

PDA Ch1 220nm

| Peak# | Ret. Time | Area | Height | Height% | Area% |
|---|---|---|---|---|---|
| 1 | 5.269 | 1535 | 442 | 0.067 | 0.027 |
| 2 | 5.523 | 5779346 | 654909 | 99.933 | 99.973 |
| Total | | 5780881 | 655351 | 100.000 | 100.000 |

$^{1}$H NMR for JAB-3312.

CAS NO.: 2245082-05-5 , CD3OD

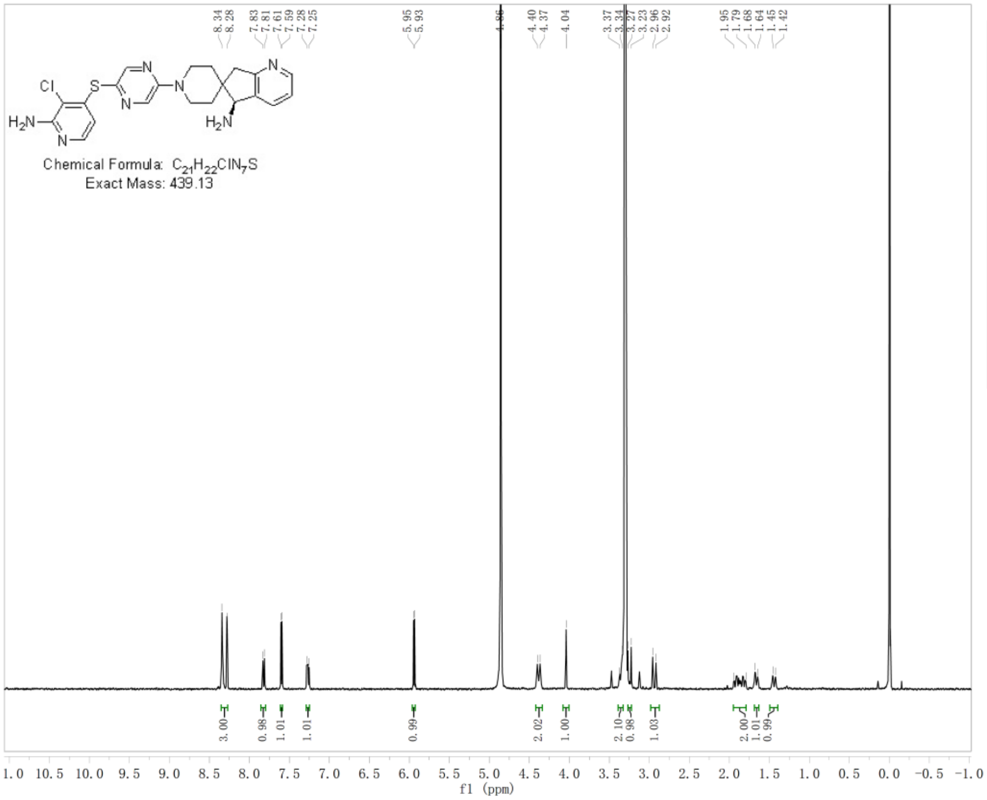


| Parameter | | Value |
|---|---|---|
| 1 | Spectrometer | Avance |
| 2 | Solvent | CD3OD_SPE |
| 3 | Temperature | 296.9 |
| 4 | Pulse Sequence | zg30 |
| 5 | Experiment | 1D |
| 6 | Number of Scans | 16 |
| 7 | Receiver Gain | 101 |
| 8 | Relaxation Delay | 1.0000 |
| 9 | Pulse Width | 8.0000 |
| 10 | Acquisition Time | 3.9977 |
| 11 | Spectrometer Frequency | 400.13 |
| 12 | Spectral Width | 8196.7 |
| 13 | Lowest Frequency | -1639.3 |
| 14 | Nucleus | 1H |
| 15 | Acquired Size | 32768 |
| 16 | Spectral Size | 65536 |

LCMS for JAB-3312.

LC/MS Report

```
File D:\data\2025-02\0206-01 09-07-35\YT-3017-TM-.D
Injection Date    : 06-Feb-25, 09:53:26             Tgt Mass(EZX) :
Sample Name       : YT-3017-TM                                 Location :
Acq. Operator     : SYSTEM                                   Inj : 1
Spec. Reported    : MS Integration                         Inj Volume : 1 ul
Acq. Method       : D:\data\2025-02\0206-01 09-07-35\C2_4min_5_95A_50_800_UV.M
Analysis Method   : D:\data\2025-02\0206-01 09-07-35\C2_4min_5_95A_50_800_UV.M
CAS NO.      : 2245082-05-5
Method Info : Instrument: LCMS005
              Mobile Phase: A: 0.1% acid B: ACN
              Gradient: 5% to 95% within 1.3min
              Flow Rate: 1.2mL/min
              Column: 4.6*50mm,3.5um YTA-RP-18
              Oven Temperature: 40C
```

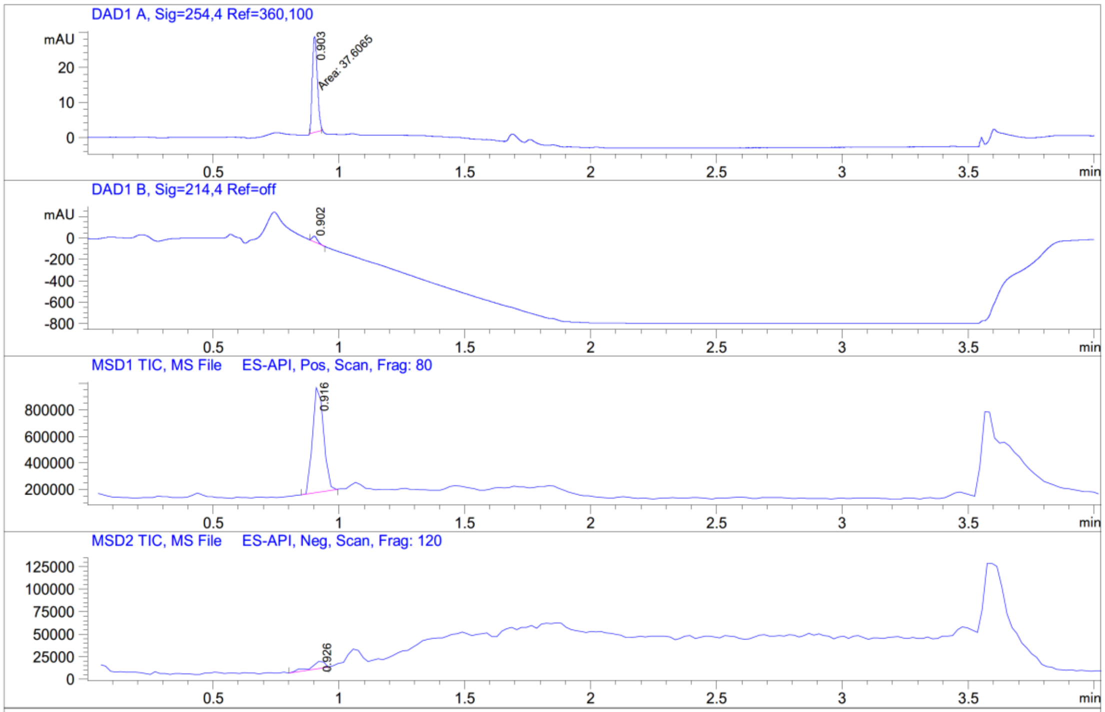


Integration Results for DAD1 A, Sig=254,4 Ref=360,100

| RetTim | Width | Area | Height | Area% |
|---|---|---|---|---|
| 0.90 | 0.02 | 37.61 | 27.34 | 100.00 |

Integration Results for DAD1 B, Sig=214,4 Ref=off

| RetTim | Width | Area | Height | Area% |
|---|---|---|---|---|
| 0.90 | 0.02 | 72.01 | 51.32 | 100.00 |

Integration Results for MSD1 TIC, MS File

| RetTim | Width | Area | Height | Area% |
|---|---|---|---|---|
| 0.92 | 0.07 | 2431256.75 | 825492.94 | 100.00 |

Integration Results for MSD2 TIC, MS File

| RetTim | Width | Area | Height | Area% |
|---|---|---|---|---|

| | | | | |
|---|---|---|---|---|
| 0.93 | 0.08 | 29168.46 | 7896.62 | 100.00 |

LC/MS Report

Ret. Time: 0.92 <<<< POSITIVE SPECTRA >>>>

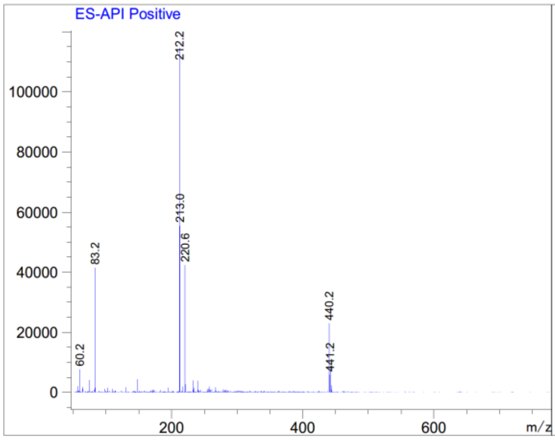


Ret. Time: 0.93 <<<< NEGATIVE SPECTRA >>>>

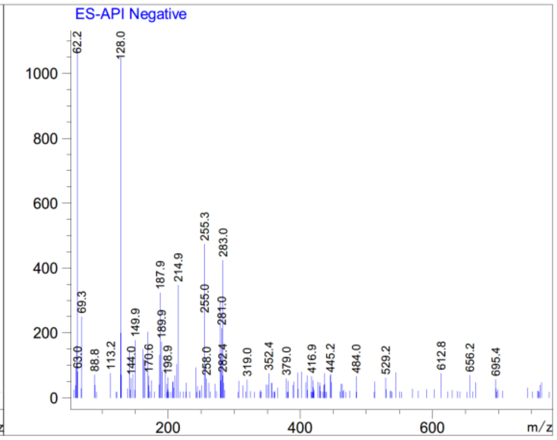

RP-HPLC for JAB-3312.

# Analysis Report

**<Sample Information>**

| | | | |
|---|---|---|---|
| CAS NO. | : 2245082-05-5 | | |
| Method Filename | : 10-90B(15MIN).lcm | | |
| Vial # | : 1-14 | Instrument | : daojin004 |
| Injection Volume | : 5 uL | | |
| Date Acquired | : 2025-02-06 10:40:17 | Acquired by | : System Administrator |

**<Chromatogram>**

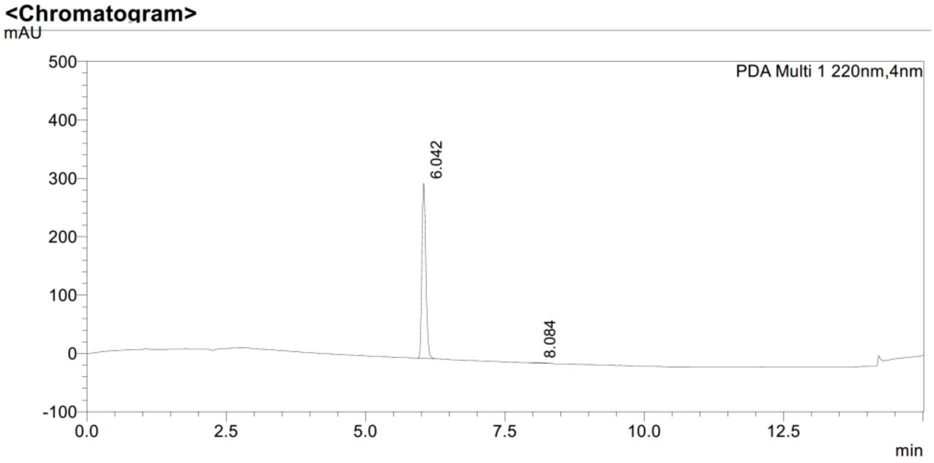


**<Peak Table>**

PDA Ch1 220nm

| Peak# | Ret. Time | Area | Height | Height% | Area% |
|---|---|---|---|---|---|
| 1 | 6.042 | 1459546 | 299726 | 99.704 | 99.316 |
| 2 | 8.084 | 10056 | 891 | 0.296 | 0.684 |
| Total | | 1469602 | 300616 | 100.000 | 100.000 |

$^{1}$H NMR for TNO155.

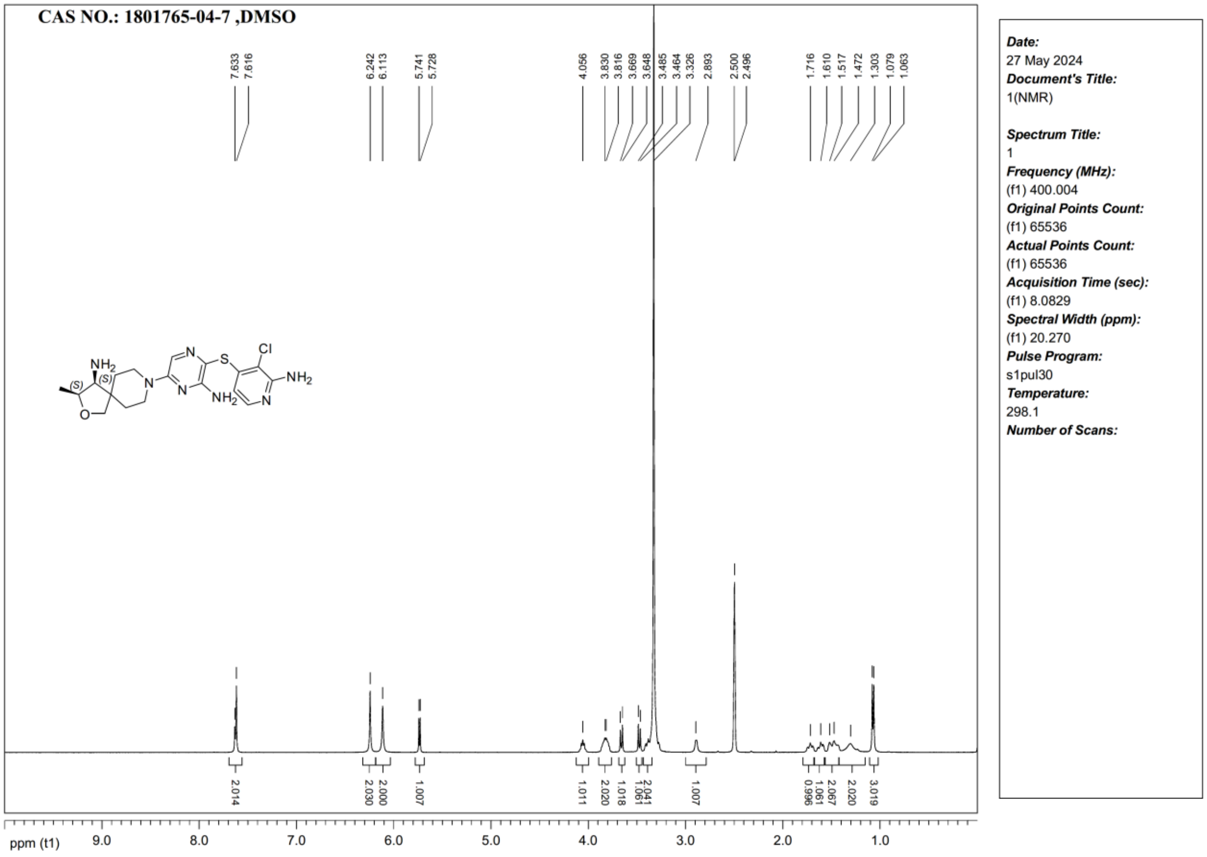

LCMS for TNO155.

MS Report from Instrument: UPLC-MS-01

```
File ..24-05-24\YWH40039-003-1-UPLC-MS-01-051762.D Tgt Mass (EZX):
Injection Date    : 24 May 24  10:12 am +0800          Seq. Line : 0
Sample Name       : YWH40039-003-1                 Location  : P1-B-06
Acq. Operator     : LY_2278                                 Inj : 1
Spec. Reported    : MS Integration                   Inj Volume : 1 ul
Acq. Method       : D:\LCMS\Methods\1-POS-UPLC2.0MIN(0.8ML)-(150-1000).M
Analysis Method   : D:\LCMS\METHODS\1-POS-UPLC2.0MIN(0.8ML)-(150-1000).M
CAS NO.        : 1801765-04-7   Walkup method: '1-POS-2MIN'  Project: LD2514  Target:
Method Info : Mobile Phase: A: water(0.01%TFA) B:ACN(0.01%TFA)
              Gradient: 5% to 95%B within 0.6 min
              Flow Rate :0.8ml/min
              Column :Poroshell 120 EC- C18, 2.1*50mm,1.9um
              Oven Temperature : 45C
```

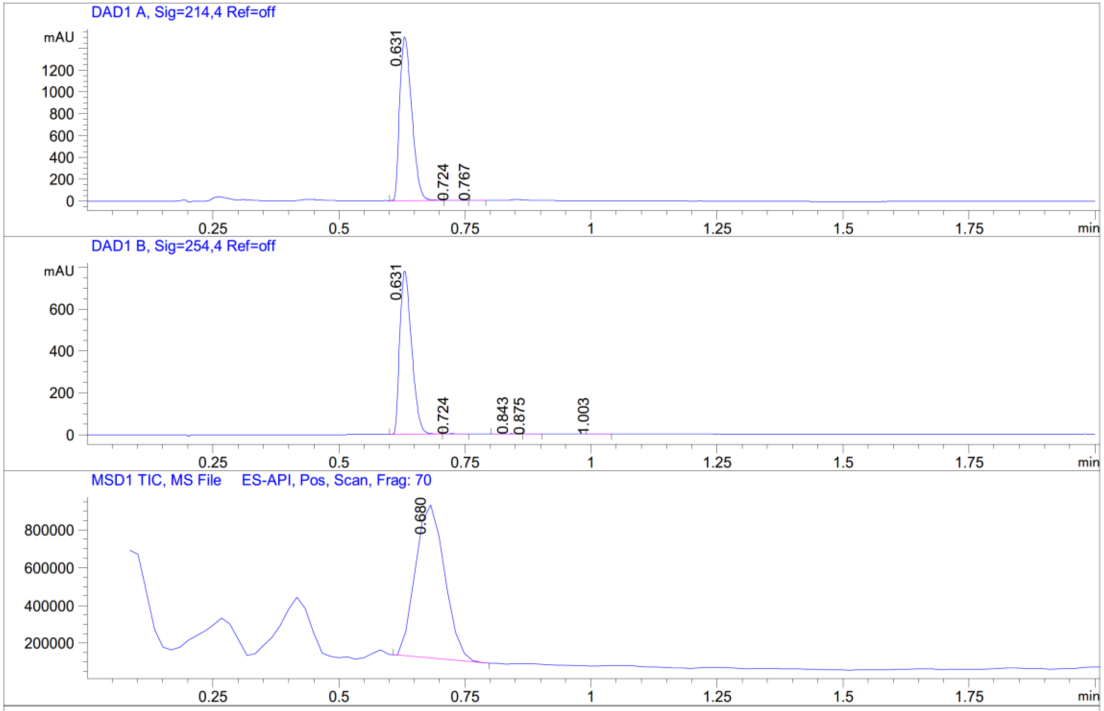


Integration Results for DAD1 A, Sig=214,4 Ref=off

| RetTim | Width | Area | Height | Area% | MS(+) |
|---|---|---|---|---|---|
| 0.63 | 0.03 | 2483.32 | 1498.38 | 99.91 | 422 |
| 0.72 | 0.02 | 1.81 | 1.41 | 0.07 | 162 |
| 0.77 | 0.01 | 0.38 | 0.42 | 0.02 | 162 |

Integration Results for DAD1 B, Sig=254,4 Ref=off

| RetTim | Width | Area | Height | Area% | MS(+) |
|---|---|---|---|---|---|
| 0.63 | 0.03 | 1262.92 | 778.76 | 99.61 | 422 |
| 0.72 | 0.02 | 2.04 | 1.64 | 0.16 | 162 |
| 0.84 | 0.02 | 1.94 | 1.48 | 0.15 | 162 |
| 0.88 | 0.02 | 0.48 | 0.44 | 0.04 | 162 |
| 1.00 | 0.01 | 0.49 | 0.51 | 0.04 | 162 |

Integration Results for MSD1 TIC, MS File

| RetTim | Width | Area | Height | Area% | MS(+) |
|---|---|---|---|---|---|
| 0.68 | 0.06 | 3281031.25 | 813333.69 | 100.00 | 422 |

MS Report from Instrument: UPLC-MS-01

Ret. Time: 0.68 <<<< POSITIVE SPECTRA >>>>

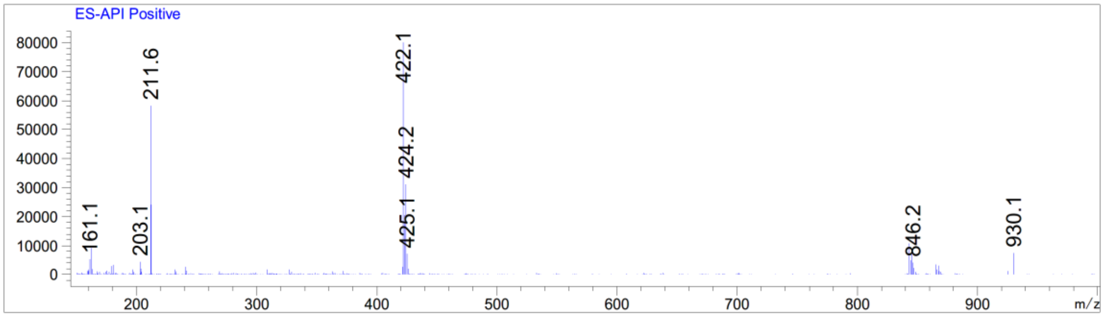

RP-HPLC for TNO155.

## SAMPLE INFORMATION

| | | | |
|---|---|---|---|
| Sample Name: | YWH40039-003-1 | CAS NO.: | 1801765-04-7 |
| Sample Type: | Unknown | Sample Set Name: | 20240524 |
| Vial: | 2:F,4 | Instrument Method Name: | HY_136173_H010P03_RP_HPLC |
| Injection #: | 1 | Processing Method: | process |
| Injection Volume: | 5.00 ul | Channel Name: | 2998 Ch1 214nm@1.2nm |
| Run Time: | 18.0 Minutes | Label | LD2514_1-20240522-A-RP-1678 |
| Date Acquired: | 5/24/2024 4:10:14 PM CST | Column Serial Number | |
| Date Processed: | 5/24/2024 4:33:19 PM CST | Processed By: | LHQ4999/QC |

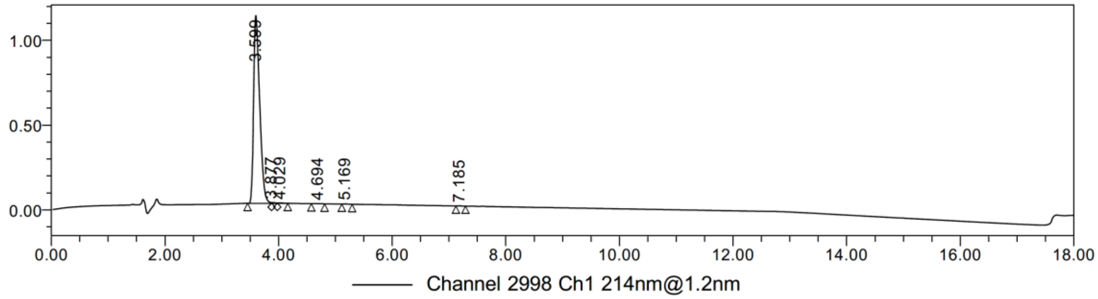


| | RT | Area | Width (sec) | Height | % Area |
|---|---|---|---|---|---|
| 1 | 3.599 | 8221283.6494 | 25.400 | 1107175.704 | 99.410 |
| 2 | 3.877 | 19505.0376 | 6.100 | 4222.996 | 0.236 |
| 3 | 4.029 | 14299.1993 | 11.000 | 2485.332 | 0.173 |
| 4 | 4.694 | 4486.5231 | 14.000 | 841.442 | 0.054 |
| 5 | 5.169 | 4236.7016 | 10.800 | 1111.791 | 0.051 |
| 6 | 7.185 | 6228.2210 | 10.200 | 1650.552 | 0.075 |

Reported by User: LHQ4999 (LHQ4999)
Report Method: Report2
Report Method ID 5726
Page: 1 of 1

Project Name: HPLC-134\2024\202405
Date Printed:
5/24/2024
4:33:47 PM PRC

**Supporting Table 1. SH2*scan* protein construct details.** Shown are the gene names, construct names, NCBI protein accession numbers, amino acid ranges and mutation status for all proteins in SH2*scan*. SH2dom.C-term. indicates a construct containing the SH2 domain closer to the C-terminus of the full-length protein when more than one SH2 domain is present in the protein. SH2dom.N-term. indicates a construct containing the SH2 domain closer to the N-terminus of the full-length protein when more than one SH2 domain is present in the protein. SH2dom1.dom2. signifies a construct containing two tandem SH2 domains (these engage a doubly phosphorylated phosphopeptide capture ligand). Nonphosphorylated constructs are harvested from protein lysates that are not treated with phosphatase inhibitors, as described in the Methods section.

| Gene Name | Construct Name | NCBI protein accession number | Amino acid range | Mutant |
|---|---|---|---|---|
| ABL1 | ABL1 | NP_009297.2 | S140/Y251 | No |
| ABL2 | ABL2 | NP_009298.1 | V165/G273 | No |
| APS (SH2B2) | APS (SH2B2) | NP_001346157.1 | E409/Q518 | No |
| BLK | BLK | NP_001706.2 | V113/A224 | No |
| BLNK | BLNK | NP_037446.1 | G317/S456 | No |
| BRK | BRK | NP_005966.1 | S75/E174 | No |
| CBL | CBL | NP_005179.2 | P47/G351 | No |
| CBLB | CBLB | NP_001308715.1 | A67/F454 | No |
| CBLC | CBLC | NP_036248.3 | M1/G323 | No |
| CISH | CISH | NP_037456.5 | K76/L275 | No |
| CRK | CRK | NP_058431.2 | D6/G124 | No |
| CRKL | CRKL | NP_005198.1 | M1/Y105 | No |
| CSK | CSK | NP_004374.1 | M80/M173 | No |
| DAPP1 | DAPP1 | NP_055210.2 | Q31/R131 | No |
| FER | FER | NP_005237.2 | K453/K552 | No |
| FES | FES | NP_001996.1 | V451/K551 | No |
| FGR | FGR | NP_005239.1 | S138/A255 | No |
| FRK | FRK | NP_002022.1 | A106/V213 | No |
| FYN | FYN | NP_002028.1 | W149/K248 | No |
| GADS | GADS | NP_004801.1 | W58/H155 | No |
| GRAP | GRAP | NP_006604.1 | P59/E151 | No |
| GRB10 | GRB10 | NP_001357938.1 | I536/R640 | No |
| GRB14 | GRB14 | NP_004481.2 | I433/R537 | No |
| GRB2 | GRB2 | NP_002077.1 | P59/I151 | No |
| GRB7 | GRB7 | NP_001025173.1 | P415/L532 | No |
| HCK | HCK | NP_002101.2 | L139/K245 | No |
| HSH2D | HSH2D | NP_116244.1 | G24/D129 | No |
| ITK | ITK | NP_005537.3 | N232/C339 | No |
| LCK | LCK | NP_005347.3 | A119/T226 | No |
| LNK (SH2B3) | LNK (SH2B3) | NP_005466.1 | D356/V465 | No |
| LYN | LYN | NP_002341.1 | S115/P229 | No |

| Gene Name | Construct Name | NCBI protein accession number | Amino acid range | Mutant |
|---|---|---|---|---|
| MATK | MATK | NP_002369.2 | L118/G216 | No |
| MIST | MIST | NP_443196.2 | W291/T422 | No |
| NCK1 | NCK1 | NP_001278928.1 | P281/S377 | No |
| NCK2 | NCK2 | NP_003572.2 | E284/Q380 | No |
| PIK3R1 | PIK3R1(SH2dom.C-term.) | NP_852664.1 | L616/R724 | No |
| PIK3R1 | PIK3R1(SH2dom.N-term.) | NP_852665.1 | G21/Q135 | No |
| PIK3R2 | PIK3R2(SH2dom.C-term.) | NP_005018.2 | D610/R717 | No |
| PIK3R2 | PIK3R2(SH2dom.N-term.) | NP_005018.2 | A317/Q432 | No |
| PIK3R3 | PIK3R3(SH2dom.C-term.) | NP_001290357.1 | N366/C477 | No |
| PIK3R3 | PIK3R3(SH2dom.N-term.) | NP_001290357.1 | S74/E188 | No |
| PLCG1 | PLCG1(SH2dom.C-term.) | NP_002651.2 | H663/E759 | No |
| PLCG1 | PLCG1(SH2dom.N-term.) | NP_002651.2 | E533/A662 | No |
| PLCG2 | PLCG2(SH2dom.C-term.) | NP_002652.2 | H641/E738 | No |
| PLCG2 | PLCG2(SH2dom.N-term.) | NP_002652.2 | E516/P640 | No |
| PTPN11 | PTPN11(SH2dom.C-term.) | NP_001317366.1 | T108/R220 | No |
| PTPN11 | PTPN11(SH2dom.N-term.) | NP_001317366.1 | M1/P107 | No |
| PTPN6 | PTPN6(SH2dom.C-term.) | NP_536859.1 | T106/R217 | No |
| PTPN6 | PTPN6(SH2dom.N-term.) | NP_536859.1 | M1/D104 | No |
| RASA1 | RASA1(SH2dom.C-term.) | NP_002881.1 | G340/Q444 | No |
| RASA1 | RASA1(SH2dom.N-term.) | NP_002881.1 | T174/D280 | No |
| SH2B (SH2B1) | SH2B (SH2B1) | NP_001139267.1 | D519/S628 | No |
| SH2D1A | SH2D1A | NP_002342.1 | M1/K104 | No |
| SH2D1B | SH2D1B | NP_444512.2 | M1/R103 | No |
| SH2D6 | SH2D6 | NP_001381392.1 | S213/P335 | No |
| SH3BP2 | SH3BP2 | NP_001139328.1 | E503/R618 | No |
| SHB | SHB | NP_003019.2 | E397/L509 | No |
| SHC1 | SHC1 | NP_001123512.1 | A481/L584 | No |
| SHC2 | SHC2 | NP_036567.2 | E479/P582 | No |
| SHC3 | SHC3 | NP_058544.3 | E492/Q594 | No |
| SHC4 | SHC4 | NP_976224.3 | K518/K630 | No |
| SHF | SHF | NP_001288097.2 | L336/L441 | No |
| SHIP1 | SHIP1 | NP_001017915.1 | M1/P112 | No |
| SHIP2 | SHIP2 | NP_001558.3 | S20/V117 | No |
| SLAP2 | SLAP2 | NP_115590.1 | A88/L193 | Np |
| SLP76 | SLP76 | NP_005556.1 | N404/T527 | No |
| SOCS1 | SOCS1 | NP_003736.1 | S60/I211 | No |
| SOCS2 | SOCS2 | NP_001257400.1 | Q32/V198 | No |
| SOCS3 | SOCS3 | NP_003946.3 | M1/L225 | No |
| SOCS4 | SOCS4 | NP_955453.1 | L274/E437 | No |
| SOCS6 | SOCS6 | NP_004223.2 | V361/S499 | No |

| Gene Name | Construct Name | NCBI protein accession number | Amino acid range | Mutant |
|---|---|---|---|---|
| SOCS7 | SOCS7 | NP_055413.2 | S451/F576 | No |
| SRC | SRC | NP_938033.1 | Q147/T250 | No |
| SRMS | SRMS | NP_543013.1 | D117/M213 | No |
| STAP1 | STAP1 | NP_036240.1 | Y168/H293 | No |
| STAP2 | STAP2 | NP_060190.2 | V138/D247 | No |
| STAT1 | STAT1 | NP_009330.1 | L136/S710 | No |
| STAT2 | STAT2 | NP_005410.1 | G130/L706 | No |
| STAT3 | STAT3 | NP_001356441.1 | G127/I722 | No |
| STAT3 | STAT3(D661Y) -nonphosphorylated | NP_001356441.1 | G127/I722 | D661Y |
| STAT3 | STAT3(E616del)-nonphosphorylated | NP_001356441.1 | G127/I722 | E616del |
| STAT3 | STAT3(N567K)-nonphosphorylated | NP_001356441.1 | G127/I722 | N567K |
| STAT3 | STAT3(N647I)-nonphosphorylated | NP_001356441.1 | G127/I722 | N647I |
| STAT3 | STAT3(S614R)-nonphosphorylated | NP_001356441.1 | G127/I722 | S614R |
| STAT3 | STAT3(Y640F) -nonphosphorylated | NP_001356441.1 | G127/I722 | Y640F |
| STAT4 | STAT4 | NP_001230764.1 | S137/S702 | No |
| STAT5A | STAT5A | NP_001275647.1 | S128/D712 | No |
| STAT5B | STAT5B | NP_036580.2 | S128/D717 | No |
| STAT5B | STAT5B(N642H) | NP_036580.2 | S128/D717 | N642H |
| STAT6 | STAT6 | NP_003144.3 | M119/Q662 | No |
| SYK | SYK(SH2dom1.dom2.) | NP_003168.2 | S9/I262 | No |
| TEC | TEC | NP_003206.2 | T235/T355 | No |
| TNS1 | TNS1 | NP_072174.3 | D1458/D1574 | No |
| TNS2 | TNS2 | NP_056134.2 | D1145/D1259 | No |
| TNS3 | TNS3 | NP_073585.8 | D1167/D1284 | No |
| TNS4 | TNS4 | NP_116254.4 | D444/E558 | No |
| TXK | TXK | NP_003319.2 | N140/S251 | No |
| VAV1 | VAV1 | NP_005419.2 | H660/T772 | No |
| VAV2 | VAV2 | NP_001127870.1 | S663/S773 | No |
| VAV3 | VAV3 | NP_006104.4 | V661/S772 | No |
| YES | YES | NP_005424.1 | P149/T262 | No |
| ZAP70 | ZAP70(SH2dom1.dom2.) | NP_001070.2 | D3/N256 | No |

**Supporting Table 2. Primary screening data for the 9 compounds tested in SH2*scan*.** Data is expressed as the mean of the normalized percent of the DMSO vehicle control, ± standard deviation. The number of independent replicates and independent experiments for the shown data are presented in **Supporting Table 3**. False positives are marked with an asterisk.

| Target | Caffeic acid-pYEEIE | CGP78850 | MN551 | Ac-pYEEIE-NH₂ | SD-36 | SI-109 | Stafib-1 | STAT6-IN-1 | STAT6-IN-3 |
|---|---|---|---|---|---|---|---|---|---|
| ABL1 | 0.0±1.4 | 3.1±1.7 | 35.7±4.3 | 106.1±10.2 | 87.4±24.0 | 131.9±19.8 | 19.9±26.7* | 0±0* | 0±46.8* |
| ABL2 | 0.7±0.4 | 6.2±0.1 | 94.6±1.6 | 110.7±17.0 | 125.7±12.9 | 122.1±5.7 | 106.0±17.7 | 98.1±4.2 | 108.3±3.5 |
| APS (SH2B2) | 2.2±0.3 | 128.1±7.6 | 94.2±3.2 | 121.8±20.8 | 140.1±6.1 | 130.4±1.6 | 64.6±31.3 | 45.3±5.2 | 66.4±17.9 |
| BLK | 0.1±0.0 | 42.1±4.6 | 115.5±14.2 | 47.8±12.5 | 70.6±5.9 | 105.3±14.7 | 91.8±35.5 | 49.1±3.4 | 67.6±11.0 |
| BLNK | 3.2±0.6 | 116.9±7.1 | 100.6±0.3 | 118.9±8.3 | 103.8±13.0 | 117.0±0.7 | 157.9±27.8 | 116.4±19.9 | 145.1±22.5 |
| BRK | 0.0±0.0 | 116.9±12.5 | 4.7±0.7* | 101.4±15.6 | 76.9±3.5 | 124.3±16.8 | 5.4±60.2* | 3.0±0.4* | 4.1±47.5* |
| CBL | 32.7±4.1 | 76.7±17.1 | 38.9±1.8 | 121.6±16.4 | 68.3±19.5 | 100.8±12.1 | 85.6±14.2 | 24.3±1.0 | 73.9±11.4 |
| CBLB | 32.7±22.6 | 122.1±4.2 | 86.8±3.2 | 116.1±4.8 | 60.9±5.8 | 128.3±5.4 | 78.9±15.9 | 36.0±0.8 | 69.0±7.6 |
| CBLC | 0.1±0.0 | 128.3±29.6 | 43.0±3.8 | 97.4±7.1 | 113.0±4.5 | 106.0±14.1 | 100.1±12.3 | 3.3±0.1 | 34.7±1.3 |
| CISH | 4.9±6.9 | 116.9±15.1 | 3.2±4.3 | 131.9±3.0 | 42.8±5.7 | 101.6±5.9 | 1.7±2.3 | 1.6±2.3 | 26.8±11.8 |
| CRK | 3.1±2.8 | 88.3±0.9 | 72.1±0.5 | 114.0±4.2 | 77.0±13.9 | 118.7±21.4 | 90.2±5.9 | 34.5±1.8 | 80.4±9.5 |
| CRKL | 42.1±6.0 | 36.4±18.5 | 202.2±6.2 | 131.3±16.6 | 119.4±31.5 | 125.3±32.5 | 120.1±104.6 | 42.4±0.9 | 102.1±19.9 |
| CSK | 103.0±4.8 | 119.6±4.5 | 105.0±7.9 | 106.4±17.0 | 97.3±14.8 | 121.2±1.9 | 111.7±5.5 | 26.3±0.8 | 65.2±5.7 |
| DAPP1 | 39.2±3.2 | 136.1±2.8 | 81.2±19.3 | 134.5±1.4 | 125.9±18.7 | 148.7±2.8 | 100.2±22.0 | 41.1±13.9 | 58.9±43.2 |
| FER | 0.8±0.8 | 135.5±7.5 | 1.1±0.2* | 37.4±14.9 | 74.4±18.6 | 107.2±24.0 | 10.8±42.1* | 8.5±0.1 | 10.7±34.0 |
| FES | 2.2±0.4 | 52.0±0.5 | 82.1±4.3 | 24.1±2.4 | 46.3±7.5 | 159.5±1.3 | 115.8±98.4 | 66.3±6.4 | 82.0±13.1 |
| FGR | 0.0±0.0 | 43.3±1.3 | 49.8±22.3 | 49.9±0.9 | 82.0±28.0 | 127.6±6.4 | 34.3±37.0* | 20.7±23.8 | 32.1±40.1 |
| FRK | 0.1±0.1 | 89.4±19.2 | 69.0±8.3 | 82.5±12.4 | 155.2±4.5 | 150.8±10.9 | 65.3±42.8 | 38.3±5.9 | 59.9±42.5 |
| FYN | 0.0±0.1 | 53.7±4.6 | 83.2±0.4 | 21.2±2.5 | 106.0±20.7 | 97.3±16.0 | 92.1±35.5 | 48.2±2.1 | 51.8±33.2 |
| GADS | 94.8±0.0 | 42.0±2.5 | 42.9±0.8 | 115.5±27.2 | 140.4±0.2 | 276.5±49.4 | 51.9±12.6 | 9.6±0.1 | 76.5±7.9 |
| GRAP | 109.9±0.3 | 0.0±0.0 | 48.7±17.4 | 112.9±3.8 | 89.2±4.6 | 140.7±11.0 | 76.2±27.0 | 52.0±15.9 | 73.5±23.6 |
| GRB10 | 0.7±0.9 | 88.5±13.4 | 61.8±16.1 | 155.2±118.9 | 111.2±9.2 | 121.7±4.4 | 78.1±12.9 | 14.6±11.0* | 58.8±17.6 |
| GRB14 | 0.0±0.0 | 45.5±1.4 | 62.1±2.4 | 56.1±7.2 | 43.2±2.9 | 110.5±5.2 | 55.1±11.6 | 24.5±4.0 | 79.3±7.7 |
| GRB2 | 73.4±9.2 | 0.0±0.0 | 103.7±0.2 | 77.1±23.9 | 92.9±1.6 | 104.9±5.4 | 79.5±8.8 | 14.7±3.1* | 72.4±21.8 |
| GRB7 | 0.2±0.1 | 0.0±0.0 | 71.3±3.5 | 123.8±0.6 | 66.9±4.6 | 134.7±14.0 | 57.9±22.2 | 47.7±4.2 | 81.2±4.6 |
| HCK | 0.0±0.3 | 60.0±12.1 | 92.7±11.3 | 82.2±11.9 | 56.4±3.2 | 91.9±27.0 | 77.0±19.3 | 48.8±5.2 | 77.4±4.3 |
| HSH2D | 80.3±18.6 | 8.1±6.6 | 3.0±0.2 | 127.4±18.6 | 72.7±14.5 | 154.5±38.0 | 27.1±53.9* | 14.6±0.1* | 19.6±29.7 |

| Target | Caffeic acid-pYEEIE | CGP78850 | MN551 | Ac-pYEEIE-$NH_2$ | SD-36 | SI-109 | Stafib-1 | STAT6-IN-1 | STAT6-IN-3 |
|---|---|---|---|---|---|---|---|---|---|
| ITK | 3.1±0.3 | 113.6±8.8 | 51.4±1.1 | 122.5±7.6 | 94.0±11.1 | 124.2±3.0 | 0±61.0* | 0.0±0.0 | 0±54.7* |
| LCK | 0.0±0.0 | 46.0±3.7 | 98.7±0.5 | 44.2±3.3 | 97.3±3.9 | 126.8±18.2 | 97.4±7.6 | 48.3±1.3 | 72.0±20.4 |
| LNK (SH2B3) | 0.4±0.1 | 79.1±5.6 | 17.7±5.5 | 72.0±12.2 | 86.6±0.0 | 81.4±6.7 | 94.8±14.4 | 6.9±3.8 | 62.0±5.7 |
| LYN | 0.0±0.0 | 40.0±0.0 | 161.3±102.3 | 38.1±2.4 | 90.9±2.3 | 140.0±1.1 | 95.6±22.4 | 73.6±54.0 | 82.3±17.1 |
| MATK | 83.8±3.5 | 153.2±7.1 | 60.4±2.8 | 197.1±10.7 | 158.6±5.1 | 163.4±8.7 | 59.9±60.2 | 6.5±1.8 | 70.3±45.8 |
| MIST | 72.9±1.4 | 101.9±2.3 | 102.3±1.8 | 55.1±77.7 | 103.5±8.6 | 103.7±3.5 | 96.8±9.3 | 88.7±5.9 | 101.6±8.1 |
| NCK1 | 0.8±0.1 | 104.4±4.8 | 67.4±5.3 | 90.1±14.4 | 106.4±10.8 | 124.1±9.7 | 53.7±39.3 | 10.6±2.6* | 85.1±10.2 |
| NCK2 | 2.8±3.1 | 111.3±23.7 | 81.5±3.0 | 74.9±14.5 | 80.0±38.4 | 121.1±35.8 | 91.4±35.1 | 66.2±8.2 | 101.7±68.4 |
| PIK3R1(SH2dom.C-term.) | 0.2±0.1 | 0.0±0.0 | 52.4±0.0 | 35.8±9.3 | 94.6±6.5 | 127.8±12.3 | 85.3±16.8 | 12.3±0.1 | 32.6±4.4 |
| PIK3R1(SH2dom.N-term.) | 0.7±0.1 | 109.2±5.7 | 99.8±1.6 | 87.7±20.5 | 123.7±16.3 | 133.8±18.4 | 88.8±24.3 | 44.8±5.0 | 85.6±12.9 |
| PIK3R2(SH2dom.C-term.) | 0.3±0.9 | 0.4±0.4* | 30.3±1.1* | 49.5±7.1 | 18.5±3.9* | 120.4±15.9 | 84.2±34.7 | 0.2±0.1 | 6.1±1.6 |
| PIK3R2(SH2dom.N-term.) | 0.0±0.2 | 141.1±24.2 | 101.2±13.4 | 74.1±10.0 | 132.1±39.4 | 130.0±27.0 | 97.2±21.5 | 48.2±5.7 | 88.4±18.2 |
| PIK3R3(SH2dom.C-term.) | 0.8±0.1 | 0.0±0.0 | 27.3±3.8 | 27.7±0.9 | 57.8±6.8 | 137.4±20.4 | 86.5±0.0 | 1.0±0.1 | 26.9±10.2 |
| PIK3R3(SH2dom.N-term.) | 0.6±0.0 | 111.3±10.3 | 97.7±10.4 | 85.7±19.1 | 110.9±7.3 | 142.0±21.5 | 105.4±23.9 | 48.3±1.7 | 85.8±16.7 |
| PLCG1(SH2dom.C-term.) | 0.0±0.0 | 96.8±4.1 | 64.1±4.5 | 37.9±3.6 | 85.7±0.8 | 98.0±1.4 | 78.8±17.8 | 1.5±2.1 | 24.7±7.0 |
| PLCG1(SH2dom.N-term.) | 0.3±0.0 | 108.7±14.6 | 62.8±3.8 | 42.1±30.1 | 91.3±4.5 | 130.4±16.7 | 82.6±20.2 | 0.3±0.1 | 32.7±14.4 |
| PLCG2(SH2dom.C-term.) | 1.1±0.1 | 104.4±2.7 | 133.0±12.6 | 90.9±15.5 | 86.3±12.4 | 98.7±6.9 | 80.4±8.2 | 7.5±0.6 | 75.3±10.0 |
| PLCG2(SH2dom.N-term.) | 1.2±0.1 | 87.2±19.7 | 109.0±67.6 | 92.3±3.3 | 73.8±3.7 | 111.7±22.4 | 72.6±27.2 | 20.7±29.3 | 51.6±17.6 |
| PTPN11(SH2dom.C-term.) | 72.6±1.1 | 127.5±18.4 | 125.6±1.4 | 133.6±0.6 | 104.4±9.8 | 136.9±12.1 | 99.1±12.9 | 2.1±0.9 | 37.9±11.0 |
| PTPN11(SH2dom.N-term.) | 92.8±10.0 | 125.2±9.1 | 85.0±0.9 | 116.6±1.5 | 89.9±0.7 | 109.2±0.7 | 79.2±4.6 | 76.7±1.3 | 73.9±34.6 |
| PTPN6(SH2dom.C-term.) | 114.5±11.0 | 130.9±0.8 | 146.7±13.0 | 110.0±19.0 | 108.2±6.9 | 119.0±0.4 | 178.8±34.7 | 45.8±7.8 | 136.2±30.6 |
| PTPN6(SH2dom.N-term.) | 117.7±19.5 | 111.7±9.8 | 101.7±14.6 | 163.9±13.1 | 87.3±4.7 | 123.4±22.8 | 27.8±4.2 | 69.7±0.0 | 77.4±15.8 |
| RASA1(SH2dom.C-term.) | 78.0±6.4 | 131.2±42.7 | 107.1±2.8 | 119.3±0.8 | 86.6±9.6 | 118.3±10.2 | 97.0±11.7 | 76.6±7.8 | 123.4±14.3 |
| RASA1(SH2dom.N-term.) | 0.1±0.0 | 47.6±2.5 | 94.2±3.0 | 121.2±7.1 | 64.7±0.3 | 122.7±19.0 | 71.3±8.8 | 50.2±0.4 | 81.7±8.5 |
| SH2B (SH2B1) | 2.3±0.4 | 125.1±1.9 | 104.8±1.6 | 126.6±2.3 | 68.4±0.4 | 148.7±6.6 | 92.7±14.3 | 22.6±6.8* | 78.1±4.8 |
| SH2D1A | 79.8±21.3 | 63.8±17.1 | 77.9±2.2 | 93.4±38.3 | 353.9±24.9 | 87.6±23.9 | 87.8±37.4 | 68.7±3.5 | 68.5±43.7 |
| SH2D1B | 12.9±2.3 | 77.3±6.6 | 63.2±6.6 | 47.1±1.3 | 99.6±1.3 | 223.4±5.4 | 62.9±9.5 | 49.6±1.2 | 46.0±22.7 |
| SH2D6 | 36.4±4.5 | 89.5±8.4 | 93.6±4.8 | 100.6±11.5 | 99.6±3.4 | 96.9±1.7 | 88.5±5.5 | 67.5±5.3 | 97.5±15.9 |
| SH3BP2 | 0.9±0.3 | 77.8±0.5 | 109.2±8.6 | 90.0±2.1 | 91.5±19.4 | 131.0±18.9 | 86.0±3.2 | 56.5±0.3 | 92.8±2.7 |
| SHB | 1.8±0.1 | 97.4±9.4 | 102.1±7.8 | 117.6±2.8 | 87.7±2.3 | 129.2±11.5 | 114.1±26.3 | 28.7±0.4 | 53.4±23.5 |
| SHC1 | 0.0±0.0 | 49.8±6.2 | 112.5±5.9 | 105.0±6.6 | 62.0±8.8 | 112.5±12.4 | 67.3±22.1 | 48.2±4.5 | 62.5±13.9 |

| Target | Caffeic acid-pYEEIE | CGP78850 | MN551 | Ac-pYEEIE-$NH_2$ | SD-36 | SI-109 | Stafib-1 | STAT6-IN-1 | STAT6-IN-3 |
|---|---|---|---|---|---|---|---|---|---|
| SHC2 | 0.3±0.1 | 100.1±8.5 | 82.9±1.7 | 138.0±4.7 | 94.9±2.3 | 131.3±12.5 | 83.4±24.4 | 47.3±3.3 | 85.2±11.3 |
| SHC3 | 0.0±0.0 | 29.2±2.8 | 91.2±1.9 | 98.9±6.8 | 74.5±0.8 | 122.5±7.3 | 87.0±14.4 | 35.3±4.8 | 50.6±6.4 |
| SHC4 | 0.0±0.0 | 108.5±7.4 | 93.0±4.4 | 119.5±2.1 | 100.3±4.5 | 135.5±12.2 | 103.0±14.0 | 77.1±7.5 | 90.2±12.0 |
| SHF | 50.6±4.3 | 77.1±2.6 | 102.9±9.4 | 60.2±17.5 | 100.0±5.2 | 78.9±3.0 | 79.7±9.4 | 43.6±7.2 | 89.5±4.6 |
| SHIP1 | 9.0±2.9 | 77.7±20.9 | 19.1±0.9* | 79.4±1.4 | 44.1±5.9 | 101.8±16.1 | 61.1±7.3 | 4.4±1.3 | 53.7±3.2 |
| SHIP2 | 0.2±0.0 | 106.5±9.3 | 31.7±18.3* | 62.5±0.8 | 67.3±2.6 | 121.0±4.5 | 56.2±25.7 | 6.9±3.9 | 36.5±29.6 |
| SLAP2 | 5.3±3.8 | 91.4±15.3 | 81.6±2.1 | 202.8±149.8 | 95.7±9.9 | 121.1±25.2 | 90.4±6.1 | 55.8±2.8 | 92.1±16.6 |
| SLP76 | 99.6±4.7 | 44.1±5.3 | 115.6±1.3 | 42.0±2.1 | 66.5±0.8 | 61.9±11.9 | 97.5±31.0 | 50.8±0.4 | 93.6±30.3 |
| SOCS1 | 1.1±0.1 | 129.4±11.7 | 38.1±7.5 | 128.1±5.7 | 132.1±8.8 | 103.3±30.8 | 156.2±34.8 | 98.5±3.5 | 95.9±35.6 |
| SOCS2 | 2.6±0.1 | 118.8±4.7 | 0.0±0.0 | 104.8±3.6 | 98.5±5.4 | 141.7±16.9 | 47.9±29.0 | 0.0±0.0 | 7.9±50.6 |
| SOCS3 | 5.3±0.3 | 115.6±4.4 | 24.4±16.1* | 149.7±16.3 | 100.4±1.2 | 153.3±21.3 | 72.7±29.0 | 39.3±22.1 | 52.6±50.6 |
| SOCS4 | 2.5±0.1 | 100.6±1.6 | 9.6±3.9 | 32.2±11.1 | 30.0±17.2 | 122.6±6.9 | 25.5±12.4 | 0.0±0.0 | 0.0±0.0 |
| SOCS6 | 8.2±0.1 | 63.3±5.3 | 65.2±0.1 | 95.6±6.2 | 57.2±3.4 | 91.9±7.4 | 96.9±12.0 | 44.1±2.1* | 90.2±12.1 |
| SOCS7 | 0.5±0.4 | 126.4±4.2 | 21.9±5.0 | 110.9±12.0 | 270.7±32.0 | 124.3±32.9 | 81.0±17.8 | 34.6±0.1 | 50.9±16.4 |
| SRC | 0.0±0.0 | 99.2±4.1 | 70.2±6.8 | 68.6±4.6 | 81.4±1.5 | 116.7±8.5 | 90.6±5.2 | 53.3±8.1 | 67.0±21.0 |
| SRMS | 0.1±0.0 | 65.3±5.9 | 1.3±0.6* | 62.7±1.1 | 102.2±1.9 | 123.4±4.6 | 8.9±56.2* | 7.6±0.5* | 8.5±53.2* |
| STAP1 | 77.9±1.0 | 113.1±2.6 | 84.3±2.8 | 101.0±11.6 | 79.9±5.8 | 103.5±11.2 | 92.0±6.8 | 65.6±5.1 | 76.9±9.2 |
| STAP2 | 58.7±7.4 | 86.0±7.4 | 143.3±67.6 | 80.1±5.7 | 36.1±5.4 | 129.5±10.5 | 78.7±36.1 | 58.4±51.4 | 56.9±15.1 |
| STAT1 | 27.1±21.3 | 71.2±2.1 | 49.8±4.1 | 69.3±10.9 | 9.1±0.0 | 0.0±1.1 | 48.2±5.4 | 0.0±0.0 | 0.7±0.6 |
| STAT2 | 0±0.0* | 27.3±0.0* | 216.9±22.6 | 53.6±1.3 | 0.1±1.3 | 100.4±0.7 | 152.6±67.8 | 35.1±16.6 | 60.3±12.5 |
| STAT3 | 102.5±22.8 | 80.9±6.4 | 67.8±17.7 | 151.4±18.8 | 0.1±0.1 | 0.4±0.1 | 66.9±10.0 | 0.0±0.0 | 0.7±0.2 |
| STAT3(D661Y)-nonphosphorylated | 108.2±9.3 | 97.4±6.3 | 171.4±3.1 | 91.4±3.8 | 0.0±0.1 | 0.6±0.2 | 157.3±49.3 | 0.0±0.0 | 4.4±3.7 |
| STAT3(E616del)-nonphosphorylated | 85.5±0.3 | 71.8±6.4 | 203.2±18.0 | 86.7±0.4 | 0.0±0.0 | 0.1±0.0 | 135.3±32.6 | 0.0±0.0 | 0.4±0.4 |
| STAT3(N567K)-nonphosphorylated | 91.0±2.3 | 78.3±2.0 | 86.5±6.2 | 94.9±0.8 | 0.1±0.0 | 0.2±0.0 | 97.5±16.8 | 0.0±0.0 | 0.2±0.3 |
| STAT3(N647I)-nonphosphorylated | 85.3±2.1 | 80.6±2.9 | 81.2±11.7 | 97.9±8.6 | 0.0±0.0 | 0.5±0.1 | 78.6±15.4 | 0.0±0.0 | 2.1±3.7 |
| STAT3(S614R)-nonphosphorylated | 81.4±6.7 | 82.4±3.2 | 95.7±8.1 | 96.3±8.9 | 1.6±0.0 | 0.1±0.0 | 87.4±4.6 | 0.0±0.0 | 0.1±0.1 |
| STAT3(Y640F)-nonphosphorylated | 130.6±28.4 | 117.6±3.3 | 49.0±5.7 | 156.0±2.6 | 0.2±0.6 | 5.8±3.7 | 75.3±26.0 | 0.0±0.0 | 2.4±6.1 |
| STAT4 | 8.8±0.4 | 91.4±14.0 | 22±0.8* | 106.6±3.0 | 1.1±0.2 | 7.3±0.1 | 57.6±15.3 | 0.0±0.0 | 0.2±0.3 |
| STAT5A | 6.2±0.4 | 84.9±21.0 | 39.0±0.8 | 94.4±4.9 | 3.4±0.4 | 100.8±9.7 | 22.0±26.6 | 0.0±0.0 | 0.0±0.0 |
| STAT5B | 19.3±2.8 | 89.6±6.1 | 49.1±7.6 | 21.8±4.7 | 5.7±0.1 | 109.2±6.0 | 0.0±0.0 | 0.0±0.0 | 0.0±0.0 |
| STAT5B(N642H) | 9.4±1.0 | 107.1±9.8 | 76.9±22.3 | 115.5±14.7 | 3.2±0.8 | 122.8±9.5 | 12.5±13.5 | 0.0±8.8 | 1.3±7.7 |
| STAT6 | 102.6±16.9 | 103.6±11.0 | 37.2±13.5 | 162.5±10.0 | 4.2±0.1 | 44.5±2.9 | 18.2±25.5* | 0.0±9.7 | 0.0±0.0 |

| Target | Caffeic acid-pYEEIE | CGP78850 | MN551 | Ac-pYEEIE-NH2 | SD-36 | SI-109 | Stafib-1 | STAT6-IN-1 | STAT6-IN-3 |
|---|---|---|---|---|---|---|---|---|---|
| SYK(SH2dom1.dom2.) | 72.6±6.4 | 85.1±0.1 | 61.9±4.0 | 126.1±1.2 | 104.6±11.7 | 121.8±4.5 | 66.1±28.1 | 61.2±11.0 | 65.2±43.9 |
| TEC | 0.1±0.0 | 126.5±0.2 | 120.4±2.4 | 119.3±6.6 | 121.1±3.0 | 135.0±2.6 | 159.2±17.1 | 75.1±3.7 | 135.7±22.9 |
| TNS1 | 25.5±1.1 | 16.4±0.3 | 133.6±23.5 | 161.1±11.7 | 112.9±1.0 | 152.5±3.2 | 131.7±25.9 | 87.6±10.0 | 88.4±24.5 |
| TNS2 | 4.8±1.3 | 16.0±0.0 | 74.1±2.1 | 104.0±5.1 | 65.4±3.3 | 126.0±5.7 | 86.0±8.8 | 55.9±0.4 | 86.8±7.1 |
| TNS3 | 1.8±0.6 | 9.8±0.1 | 48.5±7.3 | 83.2±4.0 | 57.6±6.0 | 127.9±17.2 | 77.8±9.0 | 57.0±10.1 | 55.6±12.1 |
| TNS4 | 3.1±0.7 | 5.0±1.1 | 25.9±0.1* | 61.9±0.0 | 58.1±8.9 | 109.6±29.7 | 32.7±33.4* | 14.0±3.4 | 21.6±25.3* |
| TXK | 2.7±0.3 | 74.4±2.5 | 89.1±44.1 | 109.9±5.6 | 100.7±9.7 | 118.1±15.2 | 143.5±46.3 | 87.1±5.9 | 85.4±12.9 |
| VAV1 | 1.6±0.6 | 1.8±0.4 | 108.9±7.6 | 87.2±8.1 | 116.5±3.0 | 112.0±1.7 | 106.1±16.9 | 55.3±2.6 | 67.6±32.1 |
| VAV2 | 0.5±0.1 | 50.4±7.8 | 77.8±4.8 | 49.5±2.8 | 116.7±1.5 | 119.4±0.5 | 95.8±29.1 | 79.9±1.6 | 87.5±11.3 |
| VAV3 | 0.4±0.1 | 21.7±3.7 | 113.3±17.1 | 75.8±17.0 | 82.1±3.8 | 130.2±0.9 | 101.7±15.4 | 40.3±1.3 | 76.8±21.6 |
| YES | 0.0±0.0 | 66.7±8.3 | 46.5±2.1 | 14.2±1.5 | 73.3±11.9 | 112.7±26.4 | 63.1±24.3 | 29.5±0.0 | 37.7±27.8 |
| ZAP70(SH2dom1.dom2.) | 32.3±1.6 | 91.6±5.3 | 33.5±1.9* | 134.0±4.4 | 95.6±1.3 | 129.0±1.2 | 58.0±19.5 | 51.4±0.0 | 60.7±32.9 |

**Supporting Table 3. Replicate numbers for primary screening data for the 9 compounds tested in SH2*scan*.** Independent replicate numbers (N) for each primary screening value presented in **Supporting Table 2** are shown below and were generated from at least one independent experiment.

| Target | Caffeic acid-pYEEIE | CGP78850 | MN551 | Ac-pYEEIE-$NH_2$ | SD-36 | SI-109 | Stafib-1 | STAT6-IN-1 | STAT6-IN-3 |
|---|---|---|---|---|---|---|---|---|---|
| ABL1 | 4 | 4 | 4 | 4 | 4 | 4 | 8 | 4 | 8 |
| ABL2 | 2 | 2 | 2 | 2 | 2 | 2 | 4 | 2 | 4 |
| APS (SH2B2) | 2 | 2 | 2 | 2 | 2 | 2 | 4 | 2 | 4 |
| BLK | 4 | 4 | 2 | 4 | 4 | 4 | 6 | 2 | 6 |
| BLNK | 2 | 2 | 2 | 2 | 2 | 2 | 4 | 2 | 4 |
| BRK | 2 | 2 | 2 | 2 | 2 | 2 | 4 | 2 | 4 |
| CBL | 4 | 4 | 2 | 4 | 4 | 4 | 6 | 2 | 6 |
| CBLB | 2 | 2 | 2 | 2 | 2 | 2 | 4 | 2 | 4 |
| CBLC | 2 | 2 | 2 | 2 | 2 | 2 | 4 | 2 | 4 |
| CISH | 2 | 2 | 2 | 2 | 2 | 2 | 4 | 2 | 4 |
| CRK | 2 | 2 | 2 | 2 | 2 | 2 | 4 | 2 | 4 |
| CRKL | 4 | 4 | 2 | 4 | 4 | 4 | 6 | 2 | 6 |
| CSK | 2 | 2 | 2 | 2 | 2 | 2 | 4 | 2 | 4 |
| DAPP1 | 2 | 2 | 2 | 2 | 2 | 2 | 4 | 2 | 4 |
| FER | 4 | 4 | 2 | 4 | 4 | 4 | 6 | 2 | 6 |
| FES | 2 | 2 | 4 | 2 | 2 | 2 | 4 | 4 | 4 |
| FGR | 2 | 2 | 2 | 2 | 2 | 2 | 6 | 2 | 6 |
| FRK | 2 | 2 | 2 | 2 | 2 | 2 | 4 | 2 | 4 |
| FYN | 4 | 4 | 2 | 4 | 4 | 4 | 6 | 2 | 6 |
| GADS | 2 | 2 | 2 | 2 | 2 | 2 | 4 | 2 | 4 |
| GRAP | 2 | 2 | 2 | 2 | 2 | 2 | 4 | 2 | 4 |
| GRB10 | 2 | 2 | 2 | 2 | 2 | 2 | 4 | 2 | 4 |
| GRB14 | 2 | 2 | 2 | 2 | 2 | 2 | 4 | 2 | 4 |
| GRB2 | 2 | 2 | 2 | 2 | 2 | 2 | 4 | 2 | 4 |
| GRB7 | 2 | 2 | 2 | 2 | 2 | 2 | 4 | 2 | 4 |
| HCK | 4 | 4 | 2 | 4 | 4 | 4 | 6 | 2 | 6 |
| HSH2D | 4 | 4 | 2 | 4 | 4 | 4 | 6 | 2 | 6 |
| ITK | 2 | 2 | 2 | 2 | 2 | 2 | 4 | 2 | 4 |
| LCK | 2 | 2 | 2 | 2 | 2 | 2 | 4 | 2 | 4 |
| LNK (SH2B3) | 2 | 2 | 2 | 2 | 2 | 2 | 4 | 2 | 4 |
| LYN | 2 | 2 | 2 | 2 | 2 | 2 | 4 | 2 | 4 |
| MATK | 2 | 2 | 2 | 2 | 2 | 2 | 4 | 2 | 4 |
| MIST | 2 | 2 | 2 | 2 | 2 | 2 | 4 | 2 | 4 |
| NCK1 | 2 | 2 | 2 | 2 | 2 | 2 | 4 | 2 | 4 |
| NCK2 | 4 | 4 | 2 | 4 | 4 | 4 | 6 | 2 | 6 |
| PIK3R1(SH2dom.C-term.) | 2 | 2 | 2 | 2 | 2 | 2 | 4 | 2 | 4 |

| Target | Caffeic acid-pYEEIE | CGP78850 | MN551 | Ac-pYEEIE-$NH_2$ | SD-36 | SI-109 | Stafib-1 | STAT6-IN-1 | STAT6-IN-3 |
|---|---|---|---|---|---|---|---|---|---|
| PIK3R1(SH2dom.N-term.) | 2 | 2 | 2 | 2 | 2 | 2 | 4 | 2 | 4 |
| PIK3R2(SH2dom.C-term.) | 4 | 4 | 2 | 4 | 4 | 4 | 6 | 2 | 6 |
| PIK3R2(SH2dom.N-term.) | 4 | 4 | 2 | 4 | 4 | 4 | 6 | 2 | 6 |
| PIK3R3(SH2dom.C-term.) | 2 | 2 | 2 | 2 | 2 | 2 | 4 | 2 | 4 |
| PIK3R3(SH2dom.N-term.) | 2 | 2 | 2 | 2 | 2 | 2 | 4 | 2 | 4 |
| PLCG1(SH2dom.C-term.) | 2 | 2 | 2 | 2 | 2 | 2 | 4 | 2 | 4 |
| PLCG1(SH2dom.N-term.) | 2 | 2 | 2 | 2 | 2 | 2 | 4 | 2 | 4 |
| PLCG2(SH2dom.C-term.) | 2 | 2 | 2 | 2 | 2 | 2 | 4 | 2 | 4 |
| PLCG2(SH2dom.N-term.) | 2 | 2 | 2 | 2 | 2 | 2 | 4 | 2 | 4 |
| PTPN11(SH2dom.C-term.) | 2 | 2 | 2 | 2 | 2 | 2 | 4 | 2 | 4 |
| PTPN11(SH2dom.N-term.) | 2 | 2 | 2 | 2 | 2 | 2 | 4 | 2 | 4 |
| PTPN6(SH2dom.C-term.) | 2 | 2 | 2 | 2 | 2 | 2 | 4 | 2 | 4 |
| PTPN6(SH2dom.N-term.) | 2 | 2 | 2 | 2 | 2 | 2 | 4 | 2 | 4 |
| RASA1(SH2dom.C-term.) | 2 | 2 | 2 | 2 | 2 | 2 | 4 | 2 | 4 |
| RASA1(SH2dom.N-term.) | 2 | 2 | 2 | 2 | 2 | 2 | 4 | 2 | 4 |
| SH2B (SH2B1) | 2 | 2 | 2 | 2 | 2 | 2 | 4 | 2 | 4 |
| SH2D1A | 4 | 4 | 2 | 4 | 4 | 4 | 6 | 2 | 6 |
| SH2D1B | 2 | 2 | 2 | 2 | 2 | 2 | 4 | 2 | 4 |
| SH2D6 | 2 | 2 | 2 | 2 | 2 | 2 | 4 | 2 | 4 |
| SH3BP2 | 2 | 2 | 2 | 2 | 2 | 2 | 4 | 2 | 4 |
| SHB | 2 | 2 | 2 | 2 | 2 | 2 | 4 | 2 | 4 |
| SHC1 | 2 | 2 | 2 | 2 | 2 | 2 | 4 | 2 | 4 |
| SHC2 | 2 | 2 | 2 | 2 | 2 | 2 | 4 | 2 | 4 |
| SHC3 | 2 | 2 | 2 | 2 | 2 | 2 | 4 | 2 | 4 |
| SHC4 | 2 | 2 | 2 | 2 | 2 | 2 | 4 | 2 | 4 |
| SHF | 2 | 2 | 2 | 2 | 2 | 2 | 4 | 2 | 4 |
| SHIP1 | 2 | 2 | 2 | 2 | 2 | 2 | 4 | 2 | 4 |
| SHIP2 | 2 | 2 | 2 | 2 | 2 | 2 | 6 | 2 | 6 |
| SLAP2 | 2 | 2 | 2 | 2 | 2 | 2 | 4 | 2 | 4 |
| SLP76 | 2 | 2 | 2 | 2 | 2 | 2 | 4 | 2 | 4 |
| SOCS1 | 2 | 2 | 2 | 2 | 2 | 2 | 4 | 2 | 4 |
| SOCS2 | 2 | 2 | 2 | 2 | 2 | 2 | 4 | 2 | 4 |
| SOCS3 | 2 | 2 | 4 | 2 | 2 | 2 | 6 | 4 | 6 |
| SOCS4 | 2 | 2 | 2 | 2 | 2 | 2 | 4 | 2 | 4 |
| SOCS6 | 2 | 2 | 2 | 2 | 2 | 2 | 4 | 2 | 4 |
| SOCS7 | 2 | 2 | 2 | 2 | 2 | 2 | 4 | 2 | 4 |
| SRC | 2 | 2 | 2 | 2 | 2 | 2 | 4 | 2 | 4 |
| SRMS | 2 | 2 | 2 | 2 | 2 | 2 | 4 | 2 | 4 |
| STAP1 | 2 | 2 | 2 | 2 | 2 | 2 | 4 | 2 | 4 |
| STAP2 | 2 | 2 | 2 | 2 | 2 | 2 | 4 | 2 | 4 |
| STAT1 | 2 | 2 | 2 | 2 | 2 | 2 | 4 | 2 | 4 |

| Target | Caffeic acid-pYEEIE | CGP78850 | MN551 | Ac-pYEEIE-$NH_2$ | SD-36 | SI-109 | Stafib-1 | STAT6-IN-1 | STAT6-IN-3 |
|---|---|---|---|---|---|---|---|---|---|
| STAT2 | 2 | 2 | 2 | 2 | 2 | 2 | 4 | 2 | 4 |
| STAT3 | 3 | 3 | 4 | 3 | 3 | 3 | 4 | 4 | 4 |
| STAT3(D661Y)-nonphosphorylated | 2 | 2 | 2 | 2 | 2 | 2 | 4 | 2 | 4 |
| STAT3(E616del)-nonphosphorylated | 2 | 2 | 2 | 2 | 2 | 2 | 4 | 2 | 4 |
| STAT3(N567K)-nonphosphorylated | 2 | 2 | 2 | 2 | 2 | 2 | 4 | 2 | 4 |
| STAT3(N647I)-nonphosphorylated | 2 | 2 | 2 | 2 | 2 | 2 | 4 | 2 | 4 |
| STAT3(S614R)-nonphosphorylated | 2 | 2 | 2 | 2 | 2 | 2 | 4 | 2 | 4 |
| STAT3(Y640F)-nonphosphorylated | 2 | 2 | 2 | 2 | 2 | 2 | 4 | 2 | 4 |
| STAT4 | 2 | 2 | 2 | 2 | 2 | 2 | 4 | 2 | 4 |
| STAT5A | 2 | 2 | 2 | 2 | 2 | 2 | 4 | 2 | 4 |
| STAT5B | 2 | 2 | 2 | 2 | 2 | 2 | 4 | 2 | 4 |
| STAT5B(N642H) | 2 | 2 | 6 | 2 | 2 | 2 | 8 | 6 | 8 |
| STAT6 | 2 | 2 | 4 | 2 | 2 | 2 | 4 | 4 | 4 |
| SYK(SH2dom1.dom2.) | 2 | 2 | 2 | 2 | 2 | 2 | 4 | 2 | 4 |
| TEC | 2 | 2 | 2 | 2 | 2 | 2 | 4 | 2 | 4 |
| TNS1 | 2 | 2 | 2 | 2 | 2 | 2 | 4 | 2 | 4 |
| TNS2 | 2 | 2 | 2 | 2 | 2 | 2 | 4 | 2 | 4 |
| TNS3 | 2 | 2 | 2 | 2 | 2 | 2 | 4 | 2 | 4 |
| TNS4 | 2 | 2 | 2 | 2 | 2 | 2 | 4 | 2 | 4 |
| TXK | 2 | 2 | 2 | 2 | 2 | 2 | 4 | 2 | 4 |
| VAV1 | 2 | 2 | 2 | 2 | 2 | 2 | 4 | 2 | 4 |
| VAV2 | 2 | 2 | 2 | 2 | 2 | 2 | 4 | 2 | 4 |
| VAV3 | 2 | 2 | 2 | 2 | 2 | 2 | 4 | 2 | 4 |
| YES | 2 | 2 | 2 | 2 | 2 | 2 | 4 | 2 | 4 |
| ZAP70(SH2dom1.dom2.) | 2 | 2 | 2 | 2 | 2 | 2 | 4 | 2 | 4 |

**Supporting Table 4. Dissociation constant values for the 9 compounds tested in SH2*scan*.** Mean $K_D$ values collected in this study are listed below in nM, ± standard deviation. The number of independent replicates and independent experiments for the shown data are presented in **Supporting Table 5**. False positives are colored in orange and are denoted with “no hit”. False negatives are colored in green.

| Target | Caffeic acid-pYEEIE | CGP78850 | MN551 | Ac-pYEEIE-$NH_2$ | SD-36 | SI-109 | Stafib-1 | STAT6-IN-1 | STAT6-IN-3 |
|---|---|---|---|---|---|---|---|---|---|
| ABL1 | 105±17 | 560±360 | | | | | No Hit | No Hit | No Hit |
| ABL2 | 1400±360 | 1100±120 | | | | | | | |
| APS (SH2B2) | 2300±450 | | | | | | | | |
| BLK | 1.5±0.69 | | | | | | | | |
| BLNK | 240±24 | | | | | | | | |
| BRK | 24±3.9 | | | | | | No Hit | No Hit | No Hit |
| CBL | 11000± 2200 | | | | | | | 8100±1300 | |
| CBLB | 5700±1400 | | | | | | | | |
| CBLC | 37±23 | | | | | | | 1800±490 | 2800±520 |
| CISH | 430±980 | | 46±1.4 | | | | 3900±8500 | 3600±640 | 4200±1100 |
| CRK | 2300±670 | | | | | | | 2004±5.9 | |
| CRKL | | | | | | | | | |
| CSK | | | | | | | | 3050±490 | |
| DAPP1 | | | | | | | | | |
| FER | 62±86 | | | | | | 1900±0 | 1400±71 | 8000±8700 |
| FES | 24±4.9 | | | 4200±37 | | | | | |
| FGR | 2.7±5.3 | | | | | | No Hit | 2300±550 | No Hit |
| FRK | 2.2±0.47 | | | | | | | | |
| FYN | 0.53±0.13 | | | 3300±900 | | | | | |
| GADS | | | | | | | | 2400±0 | |
| GRAP | | 3.3±0.46 | | | | | | | |
| GRB10 | 480±79 | | | | | | | No Hit | |
| GRB14 | 1200±920 | | | | | | | 3500±420 | |
| GRB2 | | 3.4±2.7 | | | | | | 6040±1400 | |
| GRB7 | 6100±320 | 5.2±0.59 | | | | | | | |
| HCK | 6.0±0.97 | | | | | | | | |
| HSH2D | | 1600±350 | 8700± 130 | | | | No Hit | No Hit | 7600±1600 |
| ITK | 570±630 | | | | | | No Hit | 10300± 4900 | |
| LCK | 3.7±0.62 | | | | | | | | |
| LNK (SH2B3) | 500±410 | | 3900±710 | | | | | 2300±140 | |
| LYN | 1.0±0.34 | | | | | | | | |

| Target | Caffeic acid-pYEEIE | CGP78850 | MN551 | Ac-pYEEIE-$NH_2$ | SD-36 | SI-109 | Stafib-1 | STAT6-IN-1 | STAT6-IN-3 |
|---|---|---|---|---|---|---|---|---|---|
| MATK | | | | | | | | 3000±490 | |
| MIST | | | | | | | | | |
| NCK1 | 97±71 | | | | | | | No Hit | |
| NCK2 | 210±59 | | | | | | | | |
| PIK3R1 (SH2dom.C-term.) | 140±100 | 150±28 | | | | | | 3400±1100 | 3000±730 |
| PIK3R1 (SH2dom.N-term.) | 140±17 | | | | | | | | |
| PIK3R2 (SH2dom.C-term.) | No Hit | No Hit | No Hit | | No Hit | | | 1000±78 | 490±80 |
| PIK3R2(SH2dom.N-term.) | 180±52 | | | | | | | | |
| PIK3R3 (SH2dom.C-term.) | 230±180 | 48±20 | 4400± 1600 | 14900± 28000 | | | | 780±42 | 1020±260 |
| PIK3R3 (SH2dom.N-term.) | 150±20 | | | | | | | | |
| PLCG1 (SH2dom.C-term.) | 14±11 | | | | | | | 2050±210 | 3800±280 |
| PLCG1 (SH2dom.N-term.) | 180±200 | | | | | | | 890±720 | 3700±330 |
| PLCG2 (SH2dom.C-term.) | 660±700 | | | | | | | 3800±500 | |
| PLCG2 (SH2dom.N-term.) | 1070±320 | | | | | | | 550±14 | |
| PTPN11 (SH2dom.C-term.) | | | | | | | | 1060±350 | |
| PTPN11 (SH2dom.N-term.) | | | | | | | | | |
| PTPN6 (SH2dom.C-term.) | | | | | | | | | |
| PTPN6 (SH2dom.N-term.) | | | | | | | 13000± 6800 | | |
| RASA1 (SH2dom.C-term.) | | | | | | | | | |
| RASA1 (SH2dom.N-term.) | 230±410 | | | | | | | | |
| SH2B (SH2B1) | 910±310 | | | | | | | | |
| SH2D1A | | | | | | | | | |
| SH2D1B | 65±14 | | | | | | | | |
| SH2D6 | | | | | | | | | |
| SH3BP2 | 1020±600 | | | | | | | | |
| SHB | 1200±530 | | | | | | | No Hit | |
| SHC1 | 320±280 | | | | | | | | |
| SHC2 | 580±330 | | | | | | | | |
| SHC3 | 130±120 | 1800±240 | | | | | | 3600±570 | |
| SHC4 | 39±6.1 | | | | | | | | |
| SHF | | | | | | | | | |
| SHIP1 | 5800±1600 | | No Hit | | | | | 4400±420 | |
| SHIP2 | 230±120 | | No Hit | | | | | 2030±420 | |
| SLAP2 | 310±100 | | | | | | | | |
| SLP76 | | | | | | | | | |
| SOCS1 | 30±6.5 | | | | | | | | |

| Target | Caffeic acid-pYEEIE | CGP78850 | MN551 | Ac-pYEEIE-$NH_2$ | SD-36 | SI-109 | Stafib-1 | STAT6-IN-1 | STAT6-IN-3 |
|---|---|---|---|---|---|---|---|---|---|
| SOCS2 | 1300±880 | | 400±35 | | | | | 1500±870 | 1010±13 |
| SOCS3 | 1800±2000 | | No Hit | | | | | | No Hit |
| SOCS4 | 1500±810 | | 64±28 | | 5900±920 | | 3900±870 | 13±0.71 | 10±5.3 |
| SOCS6 | 1900±240 | | | | | | | | |
| SOCS7 | 280±140 | | 8100±2500 | | | | | No Hit | |
| SRC | 4.8±1.3 | | | | | | | | |
| SRMS | 1.2±0.73 | | No Hit | | | | No Hit | No Hit | No Hit |
| STAP1 | | | | | | | | | |
| STAP2 | | | | | | | | | |
| STAT1 | No Hit | | | | 180±61 | 180±35 | 37000±8600 | 330±73 | 670±330 |
| STAT2 | 3.6±0.18 | No Hit | | | 450±510 | 1300±1300 | 9300±4300 | | 510±83 |
| STAT3 | | | | | 21±24 | 15±9.0 | | 180±0.01 | 370±162 |
| STAT3(D661Y)-nonphosphorylated | | | | | 24±12 | 21±6.8 | | 1000±800 | 1900±2300 |
| STAT3(E616del)-nonphosphorylated | | | | | 28±11 | 8.7±3.4 | | 140±35 | 280±120 |
| STAT3(N567K)-nonphosphorylated | | | | | 30±11 | 12±1.8 | | 190±107 | 500±200 |
| STAT3(N647I)-nonphosphorylated | | | | | 63±23 | 25±8.3 | | 800±120 | 1500±750 |
| STAT3(S614R)-nonphosphorylated | | | | | 9.7±4.1 | 5.0±0.56 | | 88±31 | 140±71 |
| STAT3(Y640F)-nonphosphorylated | | | | | 80±27 | 78±44 | | 102±44 | 95±40 |
| STAT4 | 4400±370 | | No Hit | | 120±110 | 240±106 | | 220±67 | 520±270 |
| STAT5A | 4020±1500 | | | | 1600±670 | 7030±2200 | 28000±14000 | 9.1±4.3 | 46±19 |
| STAT5B | 4200±2800 | | | | 2030±820 | 18000±4000 | 1800±560 | 16±3.2 | 69±42 |
| STAT5B(N642H) | 2400±2300 | | | | 2400±740 | 19000±8500 | 3500±1700 | 5.6±2.1 | 36±27 |
| STAT6 | | | | | 1070±740 | 1400±0.15 | 7300±2300 | 0.68±0.50 | 8.6±4.5 |
| SYK (SH2dom1.dom2.) | | | | | | | | | |
| TEC | 39±7.8 | | | | | | | | |
| TNS1 | 3800±610 | 408±160 | | | | | | | |
| TNS2 | 4300±2500 | 1200±110 | | | | | | | |
| TNS3 | 1000±210 | 620±51 | | | | | | | |
| TNS4 | 2600±740 | 508±120 | No Hit | | | | No Hit | 1800±420 | No Hit |
| TXK | 950±250 | | | | | | | | |
| VAV1 | 840±530 | 260±4.7 | | | | | | | |
| VAV2 | 58±40 | | | | | | | | |
| VAV3 | 70±12 | 860±170 | | | | | | | |
| YES | 1.0±0.47 | | | 3800±780 | | | | 9200±380 | |
| ZAP70 (SH2dom1.dom2.) | 6900±3.8 | | | | | | | | |

**Supporting Table 5. Replicate numbers for dissociation constant values for the 9 compounds tested in SH2*scan*.** Independent replicate numbers (N) for each dose-response value presented in **Supporting Table 4** are shown below and were generated from at least one independent experiment. False positives are colored in orange and false negatives are colored in green.

| Target | Caffeic acid-pYEEIE | CGP78850 | MN551 | Ac-pYEEIE-$NH_2$ | SD-36 | SI-109 | Stafib-1 | STAT6-IN-1 | STAT6-IN-3 |
|---|---|---|---|---|---|---|---|---|---|
| ABL1 | 4 | 4 | | | | | 2 | 2 | 2 |
| ABL2 | 4 | 4 | | | | | | | |
| APS (SH2B2) | 4 | | | | | | | | |
| BLK | 4 | | | | | | | | |
| BLNK | 2 | | | | | | | | |
| BRK | 4 | | | | | | 2 | 2 | 2 |
| CBL | 4 | | | | | | | 2 | |
| CBLB | 2 | | | | | | | | |
| CBLC | 10 | | | | | | | 2 | 4 |
| CISH | 38 | | 2 | | | | 20 | 2 | 4 |
| CRK | 12 | | | | | | | 2 | |
| CRKL | | | | | | | | | |
| CSK | | | | | | | | 2 | |
| DAPP1 | | | | | | | | | |
| FER | 40 | | | | | | 2 | 2 | 4 |
| FES | 4 | | | 2 | | | | | |
| FGR | 40 | | | | | | 2 | 4 | 2 |
| FRK | 4 | | | | | | | | |
| FYN | 4 | | | 2 | | | | | |
| GADS | | | | | | | | 2 | |
| GRAP | | 4 | | | | | | | |
| GRB10 | 4 | | | | | | | 2 | |
| GRB14 | 24 | | | | | | | 2 | |
| GRB2 | | 8 | | | | | | 2 | |
| GRB7 | 3 | 4 | | | | | | | |
| HCK | 4 | | | | | | | | |
| HSH2D | | 2 | 10 | | | | 2 | 2 | 2 |
| ITK | 30 | | | | | | 2 | 4 | |
| LCK | 4 | | | | | | | | |
| LNK (SH2B3) | 12 | | 2 | | | | | 2 | |
| LYN | 4 | | | | | | | | |

| Target | Caffeic acid-pYEEIE | CGP78850 | MN551 | Ac-pYEEIE-$NH_2$ | SD-36 | SI-109 | Stafib-1 | STAT6-IN-1 | STAT6-IN-3 |
|---|---|---|---|---|---|---|---|---|---|
| MATK | | | | | | | | 2 | |
| MIST | | | | | | | | | |
| NCK1 | 3 | | | | | | | 2 | |
| NCK2 | 3 | | | | | | | | |
| PIK3R1 (SH2dom.C-term.) | 29 | 3 | | | | | | 2 | 4 |
| PIK3R1 (SH2dom.N-term.) | 8 | | | | | | | | |
| PIK3R2 (SH2dom.C-term.) | 2 | 2 | 2 | | 2 | | | 2 | 4 |
| PIK3R2(SH2dom.N-term.) | 4 | | | | | | | | |
| PIK3R3 (SH2dom.C-term.) | 34 | 2 | 2 | 3 | | | | 2 | 4 |
| PIK3R3 (SH2dom.N-term.) | 4 | | | | | | | | |
| PLCG1 (SH2dom.C-term.) | 15 | | | | | | | 2 | 4 |
| PLCG1 (SH2dom.N-term.) | 21 | | | | | | | 2 | 4 |
| PLCG2 (SH2dom.C-term.) | 13 | | | | | | | 4 | |
| PLCG2 (SH2dom.N-term.) | 10 | | | | | | | 2 | |
| PTPN11 (SH2dom.C-term.) | | | | | | | | 2 | |
| PTPN11 (SH2dom.N-term.) | | | | | | | | | |
| PTPN6 (SH2dom.C-term.) | | | | | | | | | |
| PTPN6 (SH2dom.N-term.) | | | | | | | 4 | | |
| RASA1 (SH2dom.C-term.) | | | | | | | | | |
| RASA1 (SH2dom.N-term.) | 3 | | | | | | | | |
| SH2B (SH2B1) | 3 | | | | | | | | |
| SH2D1A | | | | | | | | | |
| SH2D1B | 2 | | | | | | | | |
| SH2D6 | | | | | | | | | |
| SH3BP2 | 3 | | | | | | | | |
| SHB | 10 | | | | | | | 2 | |
| SHC1 | 3 | | | | | | | | |
| SHC2 | 3 | | | | | | | | |
| SHC3 | 10 | 4 | | | | | | 2 | |
| SHC4 | 3 | | | | | | | | |
| SHF | | | | | | | | | |
| SHIP1 | 4 | | 2 | | | | | 2 | |
| SHIP2 | 12 | | 2 | | | | | 3 | |
| SLAP2 | 5 | | | | | | | | |
| SLP76 | | | | | | | | | |
| SOCS1 | 5 | | | | | | | | |

| Target | Caffeic acid-pYEEIE | CGP78850 | MN551 | Ac-pYEEIE-$NH_2$ | SD-36 | SI-109 | Stafib-1 | STAT6-IN-1 | STAT6-IN-3 |
|---|---|---|---|---|---|---|---|---|---|
| SOCS2 | 12 | | 2 | | | | | 4 | 4 |
| SOCS3 | 18 | | 2 | | | | | | 2 |
| SOCS4 | 10 | | 2 | | 2 | | 4 | 2 | 4 |
| SOCS6 | 4 | | | | | | | | |
| SOCS7 | 12 | | 2 | | | | | 2 | |
| SRC | 4 | | | | | | | | |
| SRMS | 31 | | 2 | | | | 2 | 2 | 2 |
| STAP1 | | | | | | | | | |
| STAP2 | | | | | | | | | |
| STAT1 | 2 | | | | 87 | 13 | 6 | 9 | 11 |
| STAT2 | 2 | 10 | | | 99 | 21 | 6 | | 4 |
| STAT3 | | | | | 122 | 13 | | 9 | 11 |
| STAT3(D661Y)-nonphosphorylated | | | | | 55 | 13 | | 9 | 10 |
| STAT3(E616del)-nonphosphorylated | | | | | 8 | 8 | | 9 | 11 |
| STAT3(N567K)-nonphosphorylated | | | | | 8 | 6 | | 9 | 11 |
| STAT3(N647I)-nonphosphorylated | | | | | 9 | 6 | | 9 | 10 |
| STAT3(S614R)-nonphosphorylated | | | | | 9 | 6 | | 9 | 11 |
| STAT3(Y640F)-nonphosphorylated | | | | | 48 | 10 | | 7 | 11 |
| STAT4 | 2 | | 2 | | 74 | 13 | | 9 | 11 |
| STAT5A | 2 | | | | 69 | 13 | 8 | 9 | 11 |
| STAT5B | 3 | | | | 79 | 12 | 8 | 9 | 11 |
| STAT5B(N642H) | 2 | | | | 56 | 12 | 8 | 9 | 11 |
| STAT6 | | | | | 156 | 13 | 6 | 7 | 10 |
| SYK (SH2dom1.dom2.) | | | | | | | | | |
| TEC | 4 | | | | | | | | |
| TNS1 | 4 | 10 | | | | | | | |
| TNS2 | 3 | 5 | | | | | | | |
| TNS3 | 3 | 4 | | | | | | | |
| TNS4 | 12 | 4 | 2 | | | | 2 | 2 | 4 |
| TXK | 4 | | | | | | | | |
| VAV1 | 3 | 4 | | | | | | | |
| VAV2 | 3 | | | | | | | | |
| VAV3 | 4 | 4 | | | | | | | |
| YES | 31 | | | 2 | | | | 4 | |
| ZAP70 (SH2dom1.dom2.) | 2 | | | | | | | | |

**Supporting Table 6. Literature SH2 domain binding measurements for compounds relevant to this study.** Literature values for the SH2 domain binding of compounds tested or related to those tested in this study are shown below in nM. Units reported for these measurements are listed in brackets following the value in the table. The methods used for $K_D$ or $IC_{50}$ measurement in each literature paper are listed in the table.

| Target | Caffeic acid-pYEEIE | CGP78850 | MN551 | Ac-pYEEIE | SD-36 | SI-109 | Stafib-1 | STAT6-IN-1 | STAT6-IN-3 |
|---|---|---|---|---|---|---|---|---|---|
| FYN | 84 ($IC_{50}$) | >50000 ($IC_{50}$) | | 1100 ($IC_{50}$) | | | | | |
| GRB2 | | 40 ($IC_{50}$) | | | | | | | |
| LCK | 42 ($IC_{50}$) | | | 1300 ($IC_{50}$) | | | | | |
| PIK3R1 (SH2dom.N-term.) | | >50000 ($IC_{50}$) | | | | | | | |
| PTPN11 (SH2dom.C-term.) | | >50000 ($IC_{50}$) | | | | | | | |
| PTPN11 (SH2dom.N-term.) | | >50000 ($IC_{50}$) | | | | | | | |
| SOCS2 | | | 2180 ($K_D$) | | | | | | |
| SRC | 64 ($IC_{50}$) | | | 2000 ($IC_{50}$) | | | | | |
| STAT1 | | | | | 1086 ($K_D$) | 1270 ($K_D$) | | | |
| STAT2 | | | | | 4883 ($K_D$) | 6630 ($K_D$) | | | |
| STAT3 | | | | | 44.4 ($K_D$) | 51.2 ($K_D$) | | | |
| STAT4 | | | | | 804 ($K_D$) | 1980 ($K_D$) | 3290 ($K_I$) | | |
| STAT5A | | | | | 6180 ($K_D$) | | 2420 ($K_I$) | | |
| STAT5B | | | | | 11700 ($K_D$) | | 44 ($K_I$) | | |
| STAT6 | | | | | 11325 ($K_D$) | 3370 ($K_D$) | 3470 ($K_I$) | 28 ($IC_{50}$) | 44 ($IC_{50}$) |
| **Methods Used for Binding/Activity Measurement** | Surface Plasmon Resonance (SPR) | Enzyme-Linked Immunosorbent Assay (ELISA) | Isothermal Titration Calorimetry (ITC) | Surface Plasmon Resonance (SPR) | Fluorescence Polarization (FP) and Bio-Layer Interferometry (BLI) | Fluorescence Polarization (FP) and Bio-Layer Interferometry (BLI) | Fluorescence Polarization (FP) | Fluorescence Polarization (FP) | Fluorescence Polarization (FP) |
| **References** | Park *et al.*, Bioorganic & Medicinal Chemistry Letters, 2002 | Gay *et al.*, International Journal of Cancer, 1999 | Ramachandran *et al.*, Nature Communications, 2023 | Park *et al.*, Bioorganic & Medicinal Chemistry Letters, 2002 | Bai *et al.*, Cancer Cell, 2019 | Bai *et al.*, Cancer Cell, 2019 | Elumalai *et al.*, Angewandte Chemie International Edition, 2015 | Mandal *et al.*, Journal of Medicinal Chemistry, 2015 | Mandal *et al.*, Journal of Medicinal Chemistry, 2015 |

**Supporting Table 7. The allosteric inhibitors JAB-3312 and TNO155 do not compete with the phosphopeptide capture ligand for either of the PTPN11 SH2 domain-containing constructs in SH2*scan*.** JAB-3312 and TNO155 were tested in dose response (up to a concentration of 10 µM) against the PTPN11 (N-term.SH2dom.) and PTPN11(C-term.SH2dom.) constructs of SH2scan. $K_D$s were measured over three independent experiments, each conducted in independent technical duplicate.

| Target | Compound | $K_D$, nM |
|---|---|---|
| PTPN11(SH2dom.C-term.) | JAB-3312 | >10000 |
| PTPN11(SH2dom.C-term.) | TNO155 | >10000 |
| PTPN11(SH2dom.N-term.) | JAB-3312 | >10000 |
| PTPN11(SH2dom.N-term.) | TNO155 | >10000 |

**Supporting Table 8. $K_D$ values for the nonbiotinylated forms of capture ligands and optimized bead loading percentages for each assay in SH2*scan*.** Dissociation constants for nonbiotinylated, N-acetyl (blocked) and C-amide (blocked) forms of the phosphopeptide capture ligands, as well as the optimized percent bead loading of the capture ligand, are presented for each assay in SH2*scan*. The sequence for the phosphopeptide capture ligand used in each assay is reported in the table. $K_D$s are reported as an average value, ± standard deviation, and were the result of three independent experiments, each performed in independent technical duplicate. For those assays where a $K_D$ value was not determined (ND) for the nonbiotinylated form of the phosphopeptide capture ligand due to an insufficiently large assay window, another phosphopeptide or small molecule competitor $K_D$ value is provided instead. Ahx = 6-aminohexanoic acid linker, pY = phosphotyrosine.

| Target | Capture Ligand Sequence | Percent Bead Load | Nonbiotinylated Competitor Phosphopeptide | $K_D$, nM | Alternative Phosphopeptide/Small Molecule | $K_D$, nM |
|---|---|---|---|---|---|---|
| ABL1 | Biotin-Ahx-Ahx-KEEGpYELPYNP-NH2 | 2.5 | Ac-KEEGpYELPYNP-NH2 | 28785.00±8130.27 | | |
| ABL2 | Biotin-Ahx-Ahx-SSPIpYEDAA-NH2 | 0.025 | Ac-SSPIpYEDAA-NH2 | 6203.87±584.39 | | |
| APS (SH2B2) | Biotin-Ahx-Ahx-RDIpYETDpYYR-NH2 | 25 | Ac-RDIpYETDpYYR-NH2 | ND | Ac-DDDpYDDVD-NH2 | 8781.15±971.06 |
| BLK | Biotin-Ahx-Ahx-EPQpYEEIPIYLK-NH2 | 2.5 | Ac-EPQpYEEIPIYLK-NH2 | 487.93±187.53 | | |
| BLNK | Biotin-Ahx-Ahx-LQGTpYQDVGNL-NH2 | 25 | Ac-LQGTpYQDVGNL-NH2 | ND | Ac-DDDpYDDVD-NH2 | 136.52±22.38 |
| BRK | Biotin-Ahx-Ahx-EPQpYEEIPIKQ-NH2 | 25 | Ac-EPQpYEEIPIKQ-NH2 | ND | Ac-DDDpYDDVD-NH2 | 225.99±200.03 |
| CBL | Biotin-Ahx-Ahx-TLNSDGpYTPEPA-NH2 | 25 | Ac-TLNSDGpYTPEPA-NH2 | 2204±242.49 | | |
| CBLB | Biotin-Ahx-Ahx-EDSFLQRpYSSDPT-NH2 | 2.5 | Ac-EDSFLQRpYSSDPT-NH2 | 372.9±114.34 | | |
| CBLC | Biotin-Ahx-Ahx-EDSFLQRpYSSDPT-NH2 | 2.5 | Ac-EDSFLQRpYSSDPT-NH2 | 146.46±52.01 | | |
| CISH | Biotin-Ahx-Ahx-PVPDpYTSIHIV-NH2 | 5 | Ac-PVPDpYTSIHIV-NH2 | 326.55±25.12 | | |
| CRK | Biotin-Ahx-Ahx-EPAHApYAQPQTT-NH2 | 0.25 | Ac-EPAHApYAQPQTT-NH2 | 1516.05±695.17 | | |
| CRKL | Biotin-Ahx-Ahx-EPGPpYAQPSVNT-NH2 | 0.25 | Ac-EPGPpYAQPSVNT-NH2 | 1203.75±308.66 | | |
| CSK | Biotin-Ahx-Ahx-ISAMpYSSVMK-NH2 | 0.25 | Ac-ISAMpYSSVMK-NH2 | 48.29±8.04 | | |
| DAPP1 | Biotin-Ahx-Ahx-LLPLDKDpYpYVVREPGQ-NH2 | 2.5 | Ac-LLPLDKDpYpYVVREPGQ-NH2 | 41323.33±5955.17 | | |
| FER | Biotin-Ahx-Ahx-DEpYENVD-NH2 | 0.25 | Ac-DEpYENVD-NH2 | 7773.73±2134.19 | | |
| FES | Biotin-Ahx-Ahx-VpYEPVSY-NH2 | 0.25 | Ac-VpYEPVSY-NH2 | 710.66±169.54 | | |
| FGR | Biotin-Ahx-Ahx-PEGDpYEEVLE-NH2 | 0.25 | Ac-PEGDpYEEVLE-NH2 | 1815.61±510.23 | | |
| FRK | Biotin-Ahx-Ahx-EPQpYEEIPIKQ-NH2 | 25 | Ac-EPQpYEEIPIKQ-NH2 | 6984.87±1927.80 | | |
| FYN | Biotin-Ahx-Ahx-EPQpYEEIPIYLK-NH2 | 2.5 | Ac-EPQpYEEIPIYLK-NH2 | 31.76±2.15 | | |
| GADS | Biotin-Ahx-Ahx-SDpYMNMTPRRP-NH2 | 2.5 | Ac-SDpYMNMTPRRP-NH2 | 243.83±73.30 | | |
| GRAP | Biotin-Ahx-Ahx-KPFpYVNVEF-NH2 | 0.25 | Ac-KPFpYVNVEF-NH2 | 21.29±4.83 | | |
| GRB10 | Biotin-Ahx-Ahx-DVWSpYGVTIWEL-NH2 | 50 | Ac-DVWSpYGVTIWEL-NH2 | ND | Ac-DDDpYDDVD-NH2 | 1257.14±1164.54 |
| GRB14 | Biotin-Ahx-Ahx-NPDPTIpYPVLDWN-NH2 | 2.5 | Ac-NPDPTIpYPVLDWN-NH2 | ND | Ac-PDPLpYEVMLKCWD-NH2 | 297.92±130.92 |
| GRB2 | Biotin-Ahx-Ahx-SpYINVPFTS-NH2 | 0.25 | Ac-SpYINVPFTS-NH2 | 69.35±19.40 | | |
| GRB7 | Biotin-Ahx-Ahx-PEpYVNQ-NH2 | 2.5 | Ac-PEpYVNQ-NH2 | 4474.85±557.24 | | |
| HCK | Biotin-Ahx-Ahx-EPQpYEEIPIYL-NH2 | 0.25 | Ac-EPQpYEEIPIYL-NH2 | 1437.33±474.89 | | |
| HSH2D | Biotin-Ahx-Ahx-SDpYMNMTPRRP-NH2 | 0.25 | Ac-SDpYMNMTPRRP-NH2 | 873.10±158.64 | | |
| ITK | Biotin-Ahx-Ahx-ADNDpYIIPLPD-NH2 | 25 | Ac-ADNDpYIIPLPD-NH2 | ND | Ac-DDDpYDDVD-NH2 | 1453.33±152.71 |
| LCK | Biotin-Ahx-Ahx-EPQpYEEIPIYL-NH2 | 2.5 | Ac-EPQpYEEIPIYL-NH2 | 96.96±51.62 | | |
| LNK (SH2B3) | Biotin-Ahx-Ahx-NTGDpYYDLYG-NH2 | 25 | Ac-NTGDpYYDLYG-NH2 | 3220.56±595.39 | | |
| LYN | Biotin-Ahx-Ahx-PEGDpYEEVLE-NH2 | 0.25 | Ac-PEGDpYEEVLE-NH2 | 764.98±141.38 | | |

| Target | Capture Ligand Sequence | Percent Bead Load | Nonbiotinylated Competitor Phosphopeptide | $K_D$, nM | Alternative Phosphopeptide/Small Molecule | $K_D$, nM |
|---|---|---|---|---|---|---|
| MATK | Biotin-Ahx-Ahx-NQLpYNELNLGRREEpYDVLD-NH2 | 25 | Ac-NQLpYNELNLGRREEpYDVLD-NH2 | ND | Ac-RCINpYVFFPSLKPSS-NH2 | 555.13±128.17 |
| MIST | Biotin-Ahx-Ahx-DDDpYDDVD-NH2 | 0.025 | Ac-DDDpYDDVD-NH2 | 32.88±6.70 | [redacted] | |
| NCK1 | Biotin-Ahx-Ahx-EHIpYDEVAAD-NH2 | 0.25 | Ac-EHIpYDEVAAD-NH2 | 15.33±5.07 | | |
| NCK2 | Biotin-Ahx-Ahx-EHIpYDEVAAD-NH2 | 0.025 | Ac-EHIpYDEVAAD-NH2 | ND | | |
| PIK3R1(SH2dom.C-term.) | Biotin-Ahx-Ahx-GpYVPML-OH | 0.25 | Ac-GpYVPML-OH | 4.24±0.48 | | |
| PIK3R1(SH2dom.N-term.) | Biotin-Ahx-Ahx-DMSKDESVDpYVPMLDMK-NH2 | 0.25 | Ac-DMSKDESVDpYVPMLDMK-NH2 | 41.24±9.77 | | |
| PIK3R2(SH2dom.C-term.) | Biotin-Ahx-Ahx-GpYVPML-OH | 0.025 | Ac-GpYVPML-OH | 4.82±1.95 | | |
| PIK3R2(SH2dom.N-term.) | Biotin-Ahx-Ahx-DMSKDESVDpYVPMLDMK-NH2 | 0.25 | Ac-DMSKDESVDpYVPMLDMK-NH2 | 46.70±6.22 | | |
| PIK3R3(SH2dom.C-term.) | Biotin-Ahx-Ahx-GpYVPML-OH | 0.025 | Ac-GpYVPML-OH | 2.27±0.32 | | |
| PIK3R3(SH2dom.N-term.) | Biotin-Ahx-Ahx-DMSKDESVDpYVPMLDMK-NH2 | 0.25 | Ac-DMSKDESVDpYVPMLDMK-NH2 | 78.49±26.06 | | |
| PLCG1(SH2dom.C-term.) | Biotin-Ahx-Ahx-DTEVpYESPpYADPE-NH2 | 0.25 | Ac-DTEVpYESPpYADPE-NH2 | 2907.12±2735.55 | | |
| PLCG1(SH2dom.N-term.) | Biotin-Ahx-Ahx-TSNQEpYLDLSM-NH2 | 0.25 | Ac-TSNQEpYLDLSM-NH2 | 115.75±6.69 | | |
| PLCG2(SH2dom.C-term.) | Biotin-Ahx-Ahx-DTEVpYESPpYADPE-NH2 | 25 | Ac-DTEVpYESPpYADPE-NH2 | ND | Ac-KKKKESTRSpYVILSFENNG-NH2 | 10.91±7.09 |
| PLCG2(SH2dom.N-term.) | Biotin-Ahx-Ahx-DPQRpYLVIQGDED-NH2 | 0.25 | Ac-DPQRpYLVIQGDED-NH2 | 521.43±25.43 | [redacted] | |
| PTPN11(SH2dom.C-term.) | Biotin-Ahx-Ahx-ASPEPIpYATIDFD-NH2 | 2.5 | Ac-ASPEPIpYATIDFD-NH2 | 403.23±197.77 | | |
| PTPN11(SH2dom.N-term.) | Biotin-Ahx-Ahx-ASPEPIpYATIDFD-NH2 | 0.25 | Ac-ASPEPIpYATIDFD-NH2 | 55.18±14.59 | | |
| PTPN6(SH2dom.C-term.) | Biotin-Ahx-Ahx-ATEQEITpYAELNLQK-NH2 | 0.25 | Ac-ATEQEITpYAELNLQK-NH2 | 1485.40±665.72 | | |
| PTPN6(SH2dom.N-term.) | Biotin-Ahx-Ahx-EQDPQEVTpYAQLNH-NH2 | 2.5 | Ac-EQDPQEVTpYAQLNH-NH2 | 3855.57±864.12 | | |
| RASA1(SH2dom.C-term.) | Biotin-Ahx-Ahx-GFDPSDpYAEPMDAVV-NH2 | 0.025 | Ac-GFDPSDpYAEPMDAVV-NH2 | 4.11±0.48 | | |
| RASA1(SH2dom.N-term.) | Biotin-Ahx-Ahx-EEENIpYSVPHDST-NH2 | 2.5 | Ac-EEENIpYSVPHDST-NH2 | 471.60±309.47 | | |
| SH2B (SH2B1) | Biotin-Ahx-Ahx-SPGEpYVNIEFG-NH2 | 2.5 | Ac-SPGEpYVNIEFG-NH2 | 4457.65±615.37 | | |
| SH2D1A | Biotin-Ahx-Ahx-KSLTIpYAQVQK-NH2 | 0.25 | Ac-KSLTIpYAQVQK-NH2 | 22.47±6.72 | | |
| SH2D1B | Biotin-Ahx-Ahx-KSLTIpYAQVQK-NH2 | 0.25 | Ac-KSLTIpYAQVQK-NH2 | 0.14±0.03 | | |
| SH2D6 | Caffeic acid-pYEEIE-Ahx-Ahx-K(Biotin)-NH2 | 25 | Caffeic acid-pYEEIE | 14526.08±2894.87 | | |
| SH3BP2 | Biotin-Ahx-Ahx-CSRLSIpYDNVPG-NH2 | 0.25 | Ac-CSRLSIpYDNVPG-NH2 | 209.45±10.05 | | |
| SHB | Biotin-Ahx-Ahx-SPGEpYVNIEFG-NH2 | 2.5 | Ac-SPGEpYVNIEFG-NH2 | 6031.73±1090.34 | | |
| SHC1 | Biotin-Ahx-Ahx-ENAEpYLRVA-NH2 | 2.5 | Ac-ENAEpYLRVA-NH2 | 10985.30±1264.10 | | |
| SHC2 | Biotin-Ahx-Ahx-ENAEpYLRVA-NH2 | 2.5 | Ac-ENAEpYLRVA-NH2 | 4386.58±413.34 | | |
| SHC3 | Biotin-Ahx-Ahx-ENAEpYLRVA-NH2 | 2.5 | Ac-ENAEpYLRVA-NH2 | 3777.82±471.91 | | |
| SHC4 | Biotin-Ahx-Ahx-ENAEpYLRVA-NH2 | 2.5 | Ac-ENAEpYLRVA-NH2 | 6634.48±773.04 | | |
| SHF | Caffeic acid-pYEEIE-Ahx-Ahx-K(Biotin)-NH2 | 15 | Caffeic acid-pYEEIE | 27193.17±8880.70 | Ac-KKKKESTRSpYVILSFENNG-NH2 | 1470.17±879.52 |
| SHIP1 | Biotin-Ahx-Ahx-NNVApYSYLLNR-NH2 | 0.0025 | Ac-NNVApYSYLLNR-NH2 | 241.27±71.76 | [redacted] | |
| SHIP2 | Biotin-Ahx-Ahx-NNVApYSYLLNR-NH2 | 2.5 | Ac-NNVApYSYLLNR-NH2 | 3310.62±622.43 | | |
| SLAP2 | Biotin-Ahx-Ahx-GSSDNEpYFpYVDFREY-NH2 | 25 | Ac-GSSDNEpYFpYVDFREY-NH2 | ND | Caffeic acid-pYEEIE | 313.79±104.19 |
| SLP76 | Biotin-Ahx-Ahx-DDDpYDDVD-NH2 | 0.25 | Ac-DDDpYDDVD-NH2 | 1409.86±431.81 | [redacted] | |
| SOCS1 | Biotin-Ahx-Ahx-CQVpYFTYDPYSE-NH2 | 25 | Ac-CQVpYFTYDPYSE-NH2 | 1433.23±426.43 | | |
| SOCS2 | Biotin-Ahx-Ahx-NGNNpYVYIDPT-NH2 | 25 | Ac-NGNNpYVYIDPT-NH2 | 4201.95±738.91 | | |
| SOCS3 | Biotin-Ahx-Ahx-PVPDpYTSIHIV-NH2 | 25 | Ac-PVPDpYTSIHIV-NH2 | ND | | |
| SOCS4 | Biotin-Ahx-Ahx-LLPLDKDpYpYVVREPGQ-NH2 | 2.5 | Ac-LLPLDKDpYpYVVREPGQ-NH2 | 1.91±0.50 | | |
| SOCS6 | Biotin-Ahx-Ahx-NGNNpYVYIDPT-NH2 | 0.25 | Ac-NGNNpYVYIDPT-NH2 | 20.62±4.33 | | |
| SOCS7 | Biotin-Ahx-Ahx-NGNNpYVYIDPT-NH2 | 25 | Ac-NGNNpYVYIDPT-NH2 | 11670.67±822.09 | | |

| Target | Capture Ligand Sequence | Percent Bead Load | Nonbiotinylated Competitor Phosphopeptide | $K_D$, nM | Alternative Phosphopeptide/Small Molecule | $K_D$, nM |
|---|---|---|---|---|---|---|
| SRC | Biotin-Ahx-Ahx-EPQpYEEIPIYL-NH2 | 2.5 | Ac-EPQpYEEIPIYL-NH2 | 2714.83±1948.30 | | |
| SRMS | Biotin-Ahx-Ahx-KPFpYVNVEF-NH2 | 25 | Ac-KPFpYVNVEF-NH2 | 23557.83±3034.11 | | |
| STAP1 | Biotin-Ahx-Ahx-ANSpYENVLIAK-NH2 | 25 | Ac-ANSpYENVLIAK-NH2 | 2778.88±614.57 | | |
| STAP2 | Biotin-Ahx-Ahx-ANSpYENVLIAK-NH2 | 2.5 | Ac-ANSpYENVLIAK-NH2 | 38927.50±7927.50 | | |
| STAT1 | Biotin-Ahx-Ahx-GpYDKPHVL-NH2 | 2.5 | Ac-GpYDKPHVL-NH2 | 155.22±38.19 | | |
| STAT2 | Biotin-Ahx-Ahx-RCINpYVFFPSLKPSS-NH2 | 25 | Ac-RCINpYVFFPSLKPSS-NH2 | ND | SD-36 | 454.5±509.07 |
| STAT3 | Biotin-Ahx-Ahx-GpYLPQTV-NH2 | 2.5 | Ac-pYLPQTV-NH2 | 93.54±32.81 | | |
| STAT3(D661Y)-nonphosphorylated | Biotin-Ahx-Ahx-GpYLPQTV-NH2 | 25 | Ac-pYLPQTV-NH2 | 279.25±74.60 | | |
| STAT3(E616del)-nonphosphorylated | Biotin-Ahx-Ahx-GpYLPQTV-NH2 | 25 | Ac-pYLPQTV-NH2 | 99.75±16.33 | | |
| STAT3(N567K)-nonphosphorylated | Biotin-Ahx-Ahx-GpYLPQTV-NH2 | 25 | Ac-pYLPQTV-NH2 | 150.02±13.67 | | |
| STAT3(N647I)-nonphosphorylated | Biotin-Ahx-Ahx-GpYLPQTV-NH2 | 25 | Ac-pYLPQTV-NH2 | 213.62±18.43 | | |
| STAT3(S614R)-nonphosphorylated | Biotin-Ahx-Ahx-GpYLPQTV-NH2 | 25 | Ac-pYLPQTV-NH2 | 42.35±9.29 | | |
| STAT3(Y640F)-nonphosphorylated | Biotin-Ahx-Ahx-GpYLPQTV-NH2 | 100 | Ac-pYLPQTV-NH2 | 450.74±66.51 | | |
| STAT4 | Biotin-Ahx-Ahx-SFDpYDMPHVL-NH2 | 2.5 | Ac-SFDpYDMPHVL-NH2 | 1277.93±133.28 | | |
| STAT5A | Biotin-Ahx-Ahx-GpYLVLDKW-OH | 25 | NH2-QDTpYLVLDKWL-OH | 1277.93±133.28 | | |
| STAT5B | Biotin-Ahx-Ahx-GpYLVLDKW-OH | 25 | NH2-QDTpYLVLDKWL-OH | 1015.50±179.40 | | |
| STAT5B(N642H) | Biotin-Ahx-Ahx-GpYLVLDKW-OH | 25 | NH2-QDTpYLVLDKWL-OH | 97.82±43.27 | | |
| STAT6 | Biotin-Ahx-Ahx-ApYKPFQDLI-NH2 | 25 | Ac-pYKPFQDLI-NH2 | 927.91±249.46 | | |
| SYK(SH2dom1.dom2.) | Biotin-Ahx-Ahx-NQLpYNELNLGRREEpYDVLD-NH2 | 0.25 | Ac-NQLpYNELNLGRREEpYDVLD-NH2 | 7.00±2.05 | | |
| TEC | Biotin-Ahx-Ahx-ADNDpYIIPLPD-NH2 | 25 | Ac-ADNDpYIIPLPD-NH2 | ND | Ac-EHIpYDEVAAD-NH2 | 267.44±66.31 |
| TNS1 | Biotin-Ahx-Ahx-CSRLSIpYDNVPG-NH2 | 2.5 | Ac-CSRLSIpYDNVPG-NH2 | 1348.95±529.81 | | |
| TNS2 | Biotin-Ahx-Ahx-CSRLSIpYDNVPG-NH2 | 0.25 | Ac-CSRLSIpYDNVPG-NH2 | 465.93±449.99 | | |
| TNS3 | Biotin-Ahx-Ahx-CSRLSIpYDNVPG-NH2 | 0.25 | Ac-CSRLSIpYDNVPG-NH2 | 120.34±28.89 | | |
| TNS4 | Biotin-Ahx-Ahx-CSRLSIpYDNVPG-NH2 | 0.25 | Ac-CSRLSIpYDNVPG-NH2 | 28.33±4.96 | | |
| TXK | Biotin-Ahx-Ahx-DTEVpYESPpYADPE-NH2 | 2.5 | Ac-DTEVpYESPpYADPE-NH2 | 4115.29±637.39 | | |
| VAV1 | Biotin-Ahx-Ahx-DTEVpYESPpYADPE-NH2 | 0.25 | Ac-DTEVpYESPpYADPE-NH2 | 1398.32±193.40 | | |
| VAV2 | Biotin-Ahx-Ahx-DTEVpYESPpYADPE-NH2 | 0.25 | Ac-DTEVpYESPpYADPE-NH2 | 95.13±19.87 | | |
| VAV3 | Biotin-Ahx-Ahx-DTEVpYESPpYADPE-NH2 | 0.25 | Ac-DTEVpYESPpYADPE-NH2 | 162.53±48.21 | | |
| YES | Biotin-Ahx-Ahx-EPQpYEEIPIYLK-NH2 | 2.5 | Ac-EPQpYEEIPIYLK-NH2 | 233.95±37.58 | | |
| ZAP70(SH2dom1.dom2.) | Biotin-Ahx-Ahx-NQLpYNELNLGRREEpYDVLD-NH2 | 0.025 | Ac-NQLpYNELNLGRREEpYDVLD-NH2 | 0.67±0.22 | | |
